# Use of Anti-Thrombotic Drugs and In-Hospital Mortality in Acute Aortic Dissection Patients

**DOI:** 10.3390/diagnostics12102322

**Published:** 2022-09-26

**Authors:** Kensuke Hori, Nagisa Morikawa, Eiki Tayama, Yoshihiro Fukumoto

**Affiliations:** 1 Division of Cardiovascular Medicine, Department of Internal Medicine, Kurume University School of Medicine, Kurume 830-0011, Japan; 2 Division of Cardiovascular Surgery, Department of Surgery, Kurume University School of Medicine, Kurume 830-0011, Japan

**Keywords:** acute aortic dissection, anti-thrombotic drugs, mortality, anti-coagulant, anti-platelet

## Abstract

Acute aortic dissection occurs due to a primary tear in the aortic intima, with blood from the aortic lumen entering the adjacent diseased media. In the clinical setting, practitioners often hesitate before the use of anti-thrombotic drugs in the acute phase of aortic dissection. Therefore, we examined the clinical course in patients who had already received antithrombotic therapies at the onset of acute aortic dissection, and who were given anti-thrombotic drugs in the acute phase during hospitalization. We retrospectively enrolled 685 consecutive patients with acute aortic dissection (type A/B: 454/231), who were transferred to Kurume University Hospital from 2004 to 2020. In types A and B, there were no significant differences between in-hospital mortality with or without antithrombotic therapies at the onset (14.3% vs. 16.4%, *p* = 0.66 in type A, 2.6% vs. 7.3%, *p* = 0.29 in type B). Patients in type A who survived more than a day and were treated with anti-thrombotic drugs during hospitalization had significantly lower in-hospital mortality compared with those who received no anti-thrombotic drugs in the acute phase (2.2% vs. 16.1%, *p* < 0.001), while there was no significant difference between in-hospital mortality in the two type-B groups (2.4% vs. 4.9%, *p* = 0.48). Although there were variations in response among patients with acute aortic dissection, anti-thrombotic drugs did not worsen in-hospital mortality for patients with acute aortic dissection, indicating that medical staff should not hesitate to administer anti-thrombotic drugs if indicated.

## 1. Introduction

Acute aortic dissection has poor prognosis, [1,2] frequently resulting in sudden cardiac death. There are several classifications of acute aortic dissection, among which Stanford type A is classified as “all dissections involving the ascending aorta, regardless of the site of origin”, and Stanford type B as “all dissections not involving the ascending aorta” [3]. Historically, the mortality of Stanford type A has approached 1% per hour [4] while Stanford type B has shown 10 to 25% mortality at 30 days [3]. Although diagnostic modalities and therapeutic strategies including surgical and endovascular treatment have recently advanced, mortality rates remain high [5].

Accumulated evidence has demonstrated that inflammation plays a central role in the pathogenesis of acute aortic dissection [6,7,8]. Acute aortic dissection occurs due to a primary tear in the aortic intima, with blood from the aortic lumen penetrating into diseased media [8], and thrombus often occurs in the false lumen. It has been reported that the false lumen status is associated with poor outcomes [9]. Meta-analysis has indicated that partial thrombosis in the false lumen is independently associated with poor long-term survival after acute aortic dissection, compared with complete thrombosis or patent false lumen [9]. Although the underlying mechanisms involved in the association between outcomes and false lumen status have not been fully elucidated, complete thrombus formation in the false lumen has been considered a prerequisite for healing the aorta [9]. In addition, complete thrombus may be able to relieve pressure from the tear; however, it may conversely cause organ ischemia. Furthermore, in cases of partial thrombosis in the false lumen, thrombus and platelets can modulate inflammatory reactions and immune responses [10,11]. It remains unknown how anti-thrombotic drugs affect thrombus formation in the false lumen.

Data remain scant regarding anti-thrombotic therapies in such situations, and practitioners in the clinical setting often hesitate to apply anti-thrombotic drugs in the acute phase of aortic dissection. However, anti-thrombotic drugs are occasionally required during hospitalization after acute aortic dissection, for various reasons including atrial fibrillation. Furthermore, acute aortic dissection can occur suddenly even in patients who already receive daily anti-thrombotic therapies.

Although several case reports have focused on anti-thrombotic therapy in aortic dissection [12], it is unknown whether patients with acute aortic dissection who have taken anti-thrombotic drugs have worse or comparable prognoses compared with those who have not received such treatment. Therefore, the present study aimed to examine in-hospital mortality in patients with acute aortic dissection, who were already being treated with anti-thrombotic therapies at the onset, and who were treated with anti-thrombotic agents during hospitalization in the acute phase.

## 2. Materials and Methods

### 2.1. Study Design

This study was a retrospective observational study using the database of the Coronary Care Unit/Cardiovascular Medicine, Kurume University Hospital. We enrolled patients with acute aortic dissection who were transferred to Coronary Care Unit/Cardiovascular Medicine, Kurume University Hospital, from January 2004 to December 2020. We defined “transferred“ patients as all patients who were transferred to our hospital, and “hospitalized“ as those who survived for more than a day while in our hospital. The present study was approved by the institutional review board at Kurume University (21280). Informed consent was waived due to the retrospective nature of the study.

### 2.2. Data Collection

Baseline demographic data were collected when patients arrived at the hospital, based on their medical records, including age, sex, height, body weight, waist measurement, medications including anti-thrombotic drugs (warfarin, direct oral anti-coagulants (DOAC), aspirin, clopidogrel), traditional risk factors (hypertension, glucose intolerance, diabetes mellitus, and dyslipidemia), blood pressure (BP), pulse rate, heart rate, and comorbidities (coronary artery disease, hypertensive heart disease, cardiomyopathy, valvular heart diseases, and congenital heart diseases). We recorded whether patients had already received anti-thrombotic drugs at the time of transferal, and whether those drugs were administered during hospitalization. Consciousness at arrival was evaluated using the Japan Coma Scale (JCS), which is the most commonly applied method for assessing patients’ consciousness levels: 0, alert consciousness; 1–3 (single digit), awake without any stimuli; 10–30 (double digits), arousable by some stimuli but reverting to previous state if stimulus stops; and 100–300 (triple digits), unarousable by any stimuli [13]. Coronary artery diseases included chronic stable angina, asymptomatic myocardial ischemia, acute coronary syndrome, prior myocardial infarction, prior coronary revascularization, coronary spastic angina, and nonobstructive coronary atherosclerosis. Venous thromboembolism included pulmonary thromboembolism and deep vein thrombosis. Left ventricular (LV) dysfunction was defined as LV ejection fraction <50%. The disease category “genetic and others” included Marfan syndrome, Loeys–Dietz syndrome, and Behçet’s disease. Other clinical diagnoses and medication data were obtained from medical records. All patients had their smoking and drinking habits ascertained by a questionnaire. Alcohol intake and smoking were classified as current habitual use or not. Major bleeding was defined as fatal bleeding and/or symptomatic bleeding in a critical area or organ, such as intracranial, intraspinal, intraocular, retroperitoneal, intraarticular, pericardial, or intramuscular with compartment syndrome, referring to the International Society on Thrombosis and Haemostasis [14]. Infarction included myocardial, cerebral, and peripheral, including arterial infarction of the abdomen or limbs. Myocardial infarction was diagnosed by electrocardiography (ECG) and echocardiography. Cerebral and peripheral arterial infarctions, including abdominal and limb, were diagnosed by imaging, including computed tomography scan. Details of paroxysmal atrial fibrillation were collected from medical records, having been diagnosed by ECG monitor or 12-lead ECG. Data of deaths during hospitalization were collected from medical records. All cardiovascular diseases were diagnosed by expert cardiologists.

### 2.3. Blood Sampling

Blood samples were obtained from the antecubital vein and measured at a commercial laboratory in Kurume University Hospital. Estimated glomerular filtration rate (eGFR) was calculated using the Modification of Diet in Renal Disease (MDRD) study equation modified with a Japanese coefficient [15]: eGFR (ml·min^–^^1^·1.73 m^−^^2^) = 194 × age^−^^0.287^ × serum creatinine^−^^1.094^ (if female × 0.739).

### 2.4. Outcome

Primary outcome was defined as death from any cause during hospitalization, and secondary outcomes were defined as “major bleeding”, “infarction by aortic dissection”, and “paroxysmal atrial fibrillation”, all of which were identified in the medical records.

### 2.5. Statistical Analysis

Data were presented as mean ± standard error (SE) and compared between groups using two-sided *t*-tests. Categorical variables were presented as frequency and percent proportions and were compared between groups using chi-square tests. Survival curves for deaths from all causes were estimated by the Kaplan–Meier method and compared using the log-rank test. Cox proportional hazards models were applied to estimate hazard ratios (HRs) and 95% confidence intervals (CIs) for all-cause mortality. Next, we adjusted the models for age and sex. Surgical procedure and traditional cardiovascular risk factors were considered as covariates. Statistical significance was defined as *p* < 0.05. All statistical analyses were performed using the SAS system (Release 9.4, SAS Institute, Cary, NC, USA).

## 3. Results

### 3.1. Transferred Patients

#### 3.1.1. Prognosis in Transferred Patients

**Figure 1:**
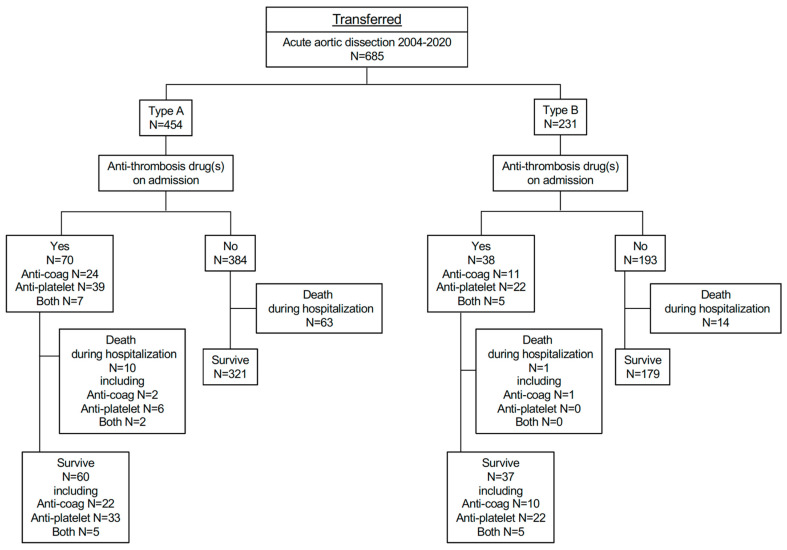
Enrollment of transferred patients with acute aortic dissection.

A total of 685 patients with acute aortic dissection were transferred to our hospital from January 2004 to December 2020 (Figure 1). There were 454 type-A and 231 type-B patients. From type A, 70 patients were treated by anti-thrombotic drugs on admission, including 24 with anti-coagulants, 39 with anti-platelet drugs, and seven with both (Figure 1). Baseline characteristics of transferred patients, including their use of anti-thrombotic drugs divided into anti-coagulants and anti-platelets, are shown in Table 1, Table 2 and Table 3.

Among 685 transferred patients, 15% of 454 type-A and 16% of 231 type-B patients had already taken anti-thrombotic drugs.

**Figure 2:**
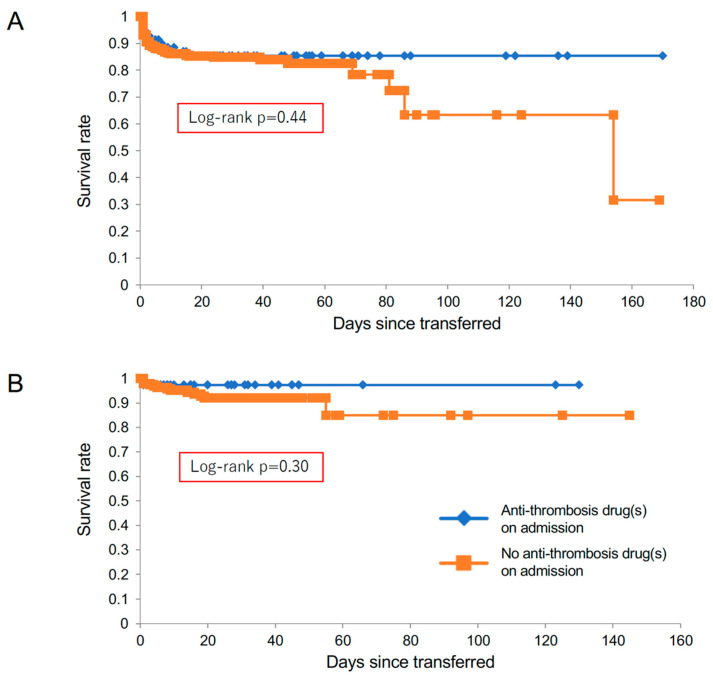
Kaplan–Meier curves of transferred patients with acute aortic dissection. There were no significant differences in survival rates between patients with and without anti-thrombotic drugs on admission, in both type A (panel (**A**)) and type B (panel (**B**)).

Type-A patients who were already being treated by anti-thrombotic drugs were significantly older, and showed a higher prevalence of previous history of atrial fibrillation, cardiovascular disease, coronary artery bypass graft, and aortic valve replacement, as well as increased use of renin-angiotensin-aldosterone system (RAAS) inhibitors (angiotensin-converting enzyme inhibitors and/or angiotensin II receptor blockers), calcium channel blockers, and β-blockers. Among them, 10 patients (14.3%) died during hospitalization (Figure 1). Of the remaining 384 patients with type-A acute aortic dissection who were not treated with anti-thrombotic drugs, 63 (16.4%) died during hospitalization (Figure 1). As indicated by the Kaplan–Meier curve, patients in type A who were treated with anti-thrombotic drugs on admission did not show worse prognosis compared with the group receiving no anti-thrombotic drugs (Figure 2A).

**Figure 3:**
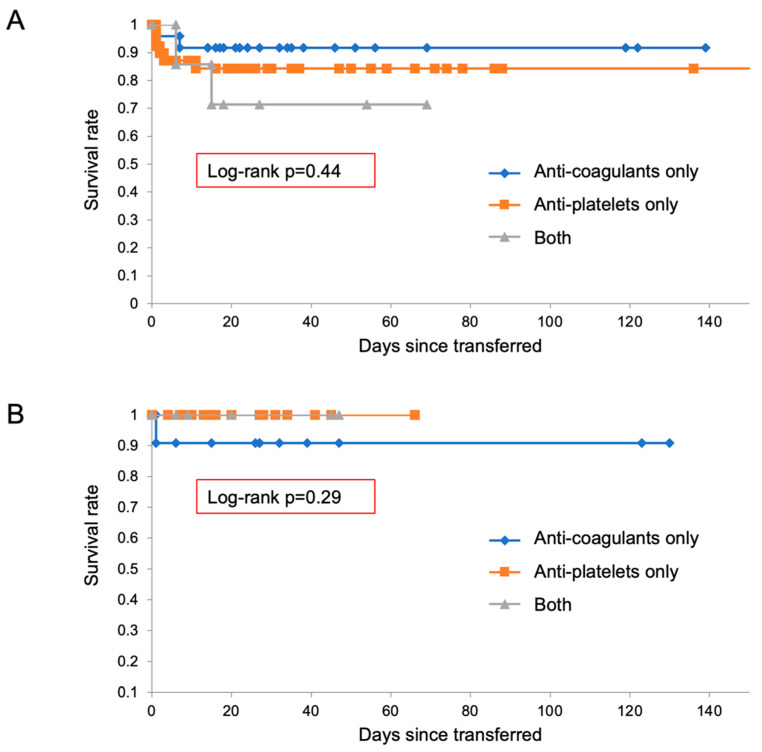
Kaplan–Meier curves of transferred patients receiving “anti-coagulants”, “anti-platelet drugs”, and “both of them”, in type A (panel (**A**)) and type B (panel (**B**)).

Furthermore, we separately analyzed the prognosis of patients receiving anti-thrombotic drugs in the categories “anti-coagulants”, “anti-platelet drugs”, and “both of them” (Figure 3). There were no significant differences in survival rates among groups receiving different anti-thrombotic drugs on admission in either type A (Figure 3A) or type B (Figure 3B).

In type B, 38 patients were treated by anti-thrombotic drugs on admission, including 11 with anti-coagulants, 22 with anti-platelet drugs, and 5 with both. This group had significantly higher prevalence of previous history of atrial fibrillation, coronary artery disease, dyslipidemia, aortic valve replacement, and coronary artery bypass graft, as well as RAAS inhibitor use (Figure 1, Table 1, Table 2 and Table 3). Among them, one patient (2.6%) died during hospitalization. Of the remaining 193 patients with type B who were not treated with anti-thrombotic drugs, 14 patients (7.3%) died during hospitalization. The Kaplan–Meier curve reveals the comparable prognosis between the two type-B groups (Figure 2B).

#### 3.1.2. Cox Proportional Hazards Regression Analysis of All-Cause Death in Transferred Patients in Type A

The Cox proportional hazards regression analysis indicated that systolic blood pressure, eGFR, history of hypertension, smoking habit, alcohol intake, complications of surgical procedures, and use of α-blockers were significantly associated with death from all causes (Table 4). According to this analysis, the use of anti-thrombotic drugs (warfarin, DOAC, aspirin, clopidogrel) was not a predictor of all cause death (Table 4).

Systolic blood pressure, eGFR, history of hypertension, smoking habit, alcohol intake, complications of surgical procedures, and use of α-blockers were significantly associated with death from all causes, after adjustment for age and sex (Table 3, Supplementary Table S1). Furthermore, the use of anti-thrombotic drugs (warfarin, DOAC, aspirin, clopidogrel) was not a predictor of all-cause death (Table 5, Supplementary Table S1), and this finding remained consistent in the fully adjusted models (Supplementary Table S1).

#### 3.1.3. Cox Proportional Hazards Regression Analysis of All-Cause Death in Transferred Patients in Type B

In the Cox proportional hazards regression analysis of all-cause death, systolic blood pressure, heart rate, eGFR, history of atrial fibrillation, complications of surgical procedures, and use of diuretics were significantly associated with death from all causes (Table 4). It was also revealed in in this analysis that the use of anti-thrombotic drugs (warfarin, DOAC, aspirin, clopidogrel) was not a predictor of all-cause death (Table 4).

After adjustment for age and sex, systolic blood pressure, heart rate, eGFR, history of atrial fibrillation, complications of surgical procedures, and use of diuretics were significantly associated with death from all causes (Table 5, Supplementary Table S2), while the use of anti-thrombotic drugs was not a predictor of all-cause death, even when the association was adjusted for other confounders (Table 5, Supplementary Table S2).

### 3.2. Hospitalized Patients

#### 3.2.1. Prognosis in Hospitalized Patients

**Figure 4:**
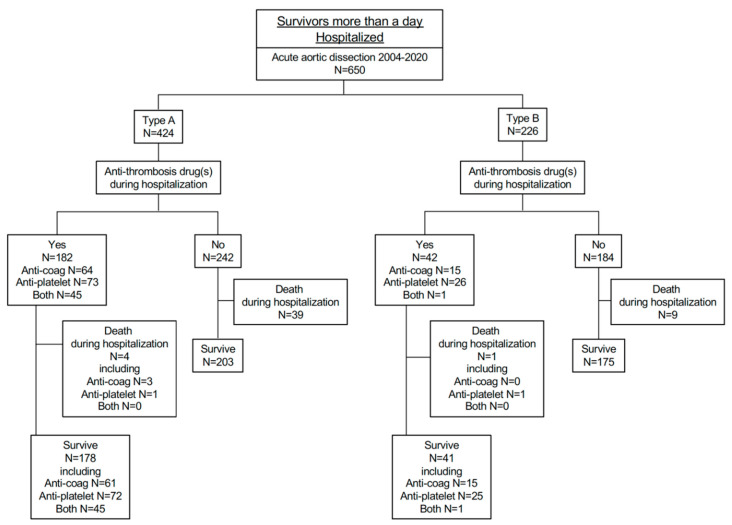
Enrollment of hospitalized patients with acute aortic dissection.

A total of 650 patients with acute aortic dissection survived for more than a day when hospitalized (Figure 4). This figure included 424 type-A and 226 type-B patients. In type A, 182 patients were treated by anti-thrombotic drugs during hospitalization, including 64 with anti-coagulants, 73 with anti-platelet drugs, and 45 with both. Baseline characteristics of hospitalized patients, including their anti-thrombotic drug treatment by anti-coagulants or anti-platelet agents, are shown in Table 6, Table 7 and Table 8. Type-A patients had a significantly higher prevalence of atrial fibrillation in association with anti-coagulant therapy, and genetic and other aortic diseases including Marfan syndrome, Loeys-Dietz syndrome, and Behçet’s disease in association with anti-coagulant and anti-platelet therapies (Table 6, Table 7 and Table 8).

Among 650 hospitalized patients, 43% of 424 type-A and 19% of 225 type-B patients were treated with anti-thrombotic drugs during hospitalization.

**Figure 5:**
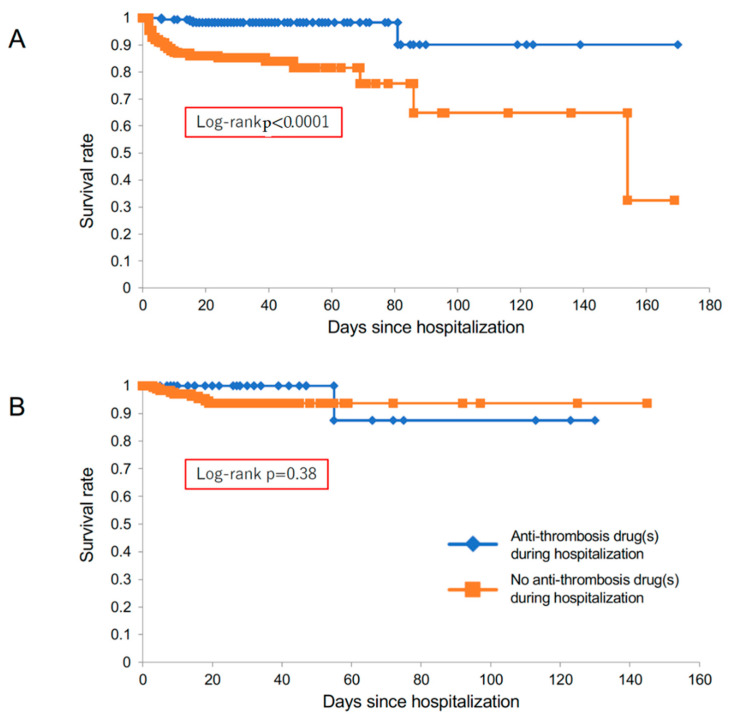
Kaplan–Meier curves of hospitalized patients with acute aortic dissection, in type A (panel (**A**)) and type B (panel (**B**)).

Among them, four patients (2.2%) died during hospitalization, and 178 (97.8% were discharged (Figure 4). Of the remaining 242 type-A patients who were not treated with anti-thrombotic drugs, 39 (16.1%) died and 203 (83.9%) were discharged (Figure 4). The Kaplan–Meier curve shows the significantly better prognosis in patients in type A treated with anti-thrombotic drugs (Figure 5A).

Survival rate was significantly better in the anti-thrombotic drug group than the other group in type A (Figure 5A), while in type B there was no significant difference between those with and without anti-thrombotic drug treatment during hospitalization (Figure 5B).

Of 226 type-B patients, 42 were treated with anti-thrombotic drugs, including 15 with anti-coagulants, 26 with anti-platelet drugs, and a single patient who received both (Figure 4). Altogether, this group had significantly higher prevalence of atrial fibrillation, coronary artery disease, aortic valve replacement, and coronary artery bypass graft (Table 6, Table 7 and Table 8). Among them, one patient (2.4%) died, and the remainder were discharged. Among the 184 type-B patients who were not treated by anti-thrombotic drugs, nine of them (4.9%) died during hospitalization (Figure 4). As indicated in the Kaplan–Meier curve, there were no significant differences in prognosis among patients in type B (Figure 5B).

**Figure 6:**
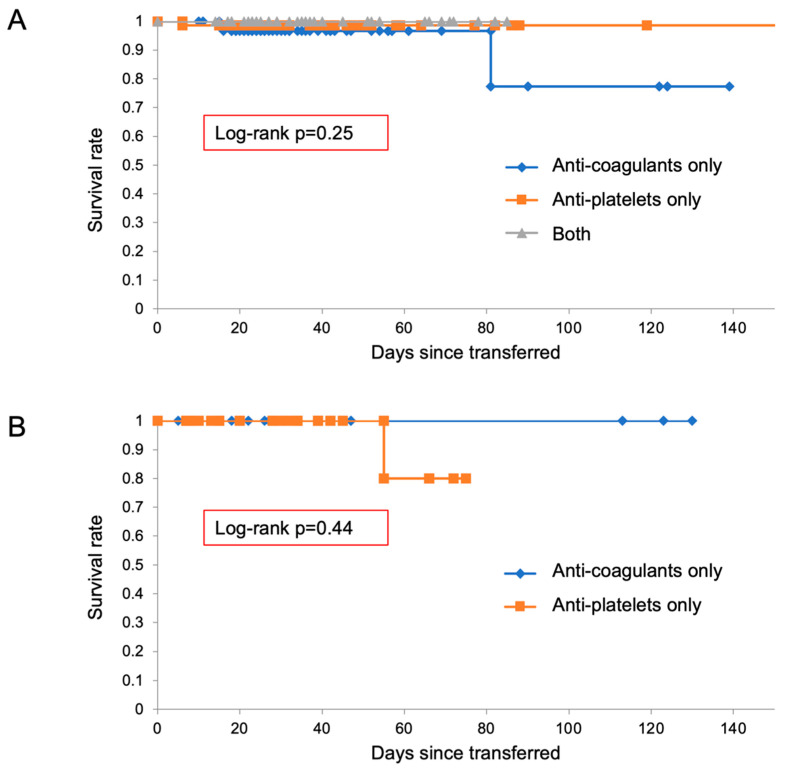
Kaplan–Meier curves of transferred patients receiving “anti-coagulants”, “anti-platelet drugs”, and “both of them”, in type A (panel (**A**)) and type B (panel (**B**)).

Again, we separately analyzed the prognosis of patients receiving treatment with anti-thrombotic drugs, according to whether they received “anti-coagulants”, “anti-platelet drugs”, or “both of them” (Figure 6). There were no significant differences in survival rates among these groups in either type A (Figure 6A) or type B (Figure 6B).

In Figure 6B, a third survival curve was not obtained because the group receiving “both” treatments contained only one patient, who survived for the whole observational period.

#### 3.2.2. Cox Proportional Hazards Regression Analysis of All-Cause Death in Hospitalized Patients in Type A

In the analysis of deaths from all causes, systolic blood pressure, eGFR, complications of surgical procedures, and use of α-blockers and anti-thrombotic drugs were significantly associated with all-cause death (Table 9). The use of anti-thrombotic drugs (warfarin, DOAC, aspirin, clopidogrel) significantly reduced the hazard ratio (Table 9).

Systolic blood pressure, eGFR, complications of surgical procedures, and use of α-blockers were significantly associated with all-cause death, after adjustment for age and sex (Table 10, Supplementary Table S3). According to this analysis and the fully adjusted models, the use of anti-thrombotic drugs was also a significant and strong predictor of reduced frequency of deaths from all causes (Table 10, Supplementary Table S3).

#### 3.2.3. Cox Proportional Hazards Regression Analysis of All-Cause Death in Hospitalized Patients in Type B

In the analysis of deaths from all causes, it was found that heart rate, history of atrial fibrillation, and complications of surgical procedures were significantly associated with all-cause death (Table 9). In this analysis, the use of anti-thrombotic drugs was not found to be a predictor of all-cause death (Table 9).

History of atrial fibrillation and complications of surgical procedures were significantly associated with all-cause death, after adjustment for age and sex (Table 10, Supplementary Table S4). The use of anti-thrombotic drugs was not a predictor of all-cause death, regardless of cardiovascular risk factors or other confounders (Table 10, Supplementary Table S4).

#### 3.2.4. Analyses of Newly Administered Anti-Thrombotic Agents during Hospitalization

Anti-coagulants were newly administered in 92 patients, while 33 patients had already taken anti-coagulants before admission and continued to do so during hospitalization. Anti-platelet agents were newly administered in 104 patients, while 41 patients had already taken anti-platelet drugs before admission and continued to do so during hospitalization (Table 11). In type A, newly administered anti-thrombotic agents corresponded with significantly better survival rates (Table 11 and Supplementary Table S5).

## 4. Discussion

This is the first report to demonstrate that the use of anti-thrombotic drugs did not worsen prognosis in patients with acute aortic dissection, although there was considerable heterogeneity between the two groups with and without anti-thrombotic drugs. In the anti-thrombotic drug group, higher prevalence of previous history of atrial fibrillation and cardiovascular disease was observed, which might have worsened prognosis. Although certain hidden conditions might have affected the findings, the use of anti-thrombotic drugs during hospitalization showed significantly better prognosis in type A, where it was observed even after adjustment for age and sex. Probably because more attention was paid to surgical repair in patients who received anti-thrombotic drugs, those drugs did not worsen the prognosis in acute aortic dissection. In particular, type A is difficult for cardiac surgeons to treat with surgical procedures. However, due to the efforts of cardiac surgeons, the use of anti-platelet drugs or anti-coagulants did not worsen prognosis after surgery in type A.

### 4.1. Initial Medical Therapy in Acute Aortic Dissection

Initial medical therapy should stabilize patients, control pain, and lower blood pressure, to prevent further propagation of the dissection and to reduce the risk of aortic rupture [16]. The present study demonstrated that anti-thrombotic drugs do not worsen the prognosis; however, the new introduction of anti-thrombotic drugs is not recommended before appropriate surgical consultation or transfer of patients to comprehensive aortic centers [16].

### 4.2. Medical Management during Hospitalization

Blood pressure management is vital for patients with acute aortic dissection [16]. β-adrenergic blockade reduces heart rate and blood pressure, and provides anti-impulse therapy to decrease aortic wall stress [17]. In addition to appropriate blood pressure management, other therapeutic medical strategies are required. Although the present study has demonstrated that prognosis was comparable between patient groups with and without anti-thrombotic drugs during hospitalization with type-B aortic dissection, the anti-thrombotic drug group showed significantly better prognosis among patients with type-A aortic dissection. Even after separation into groups receiving anti-coagulants or anti-platelet agents, both type-A groups showed significantly better prognosis. However, aortic dissection can vary greatly in its presentation, thus reducing the effectiveness of comparison between the two groups with and without anti-thrombotic drugs. Furthermore, anti-thrombotic drugs might not have been administered in severely ill patients, even when they were indicated, while such drugs may have been given to stable patients. In the present study, we were not able to exclude confounding by indication.

### 4.3. Long-Term Medical Management

Survival rate has been reported between 52% and 94% at 1 year and between 45% and 88% at 5 years in type A [18], and between 56% and 92 % at 1 year and between 48% and 82% at 5 years in type B [19]. Long-term management of acute aortic dissection should be carried out to prevent late-phase complications, including aneurysmal enlargement and aortic rupture. Poorly controlled hypertension has been reported to increase late morbidity and mortality [20,21]. Thus, it is necessary to control blood pressure, to screen patients and relatives for heritable disorders associated with aortic dissection, to complete serial imaging of the aorta, to encourage lifestyle modifications, and to provide education [18,22].

### 4.4. Limitations

The present study has several limitations. First, it was an observational retrospective cohort study from a single center. Anti-thrombotic drugs were not administered for aortic dissection, but for other cardiovascular diseases such as coronary artery diseases or atrial fibrillation. Because the preoperative use of a drug reflects the existence of a disease (i.e., DOAC for atrial fibrillation) which might affect the outcome, it is impossible to assess whether effects on outcomes are due to the drug or the disease. This study did not aim to show the superiority of anti-thrombotic drugs in patients with acute aortic dissection. A further limitation was that the enrolled patients were hospitalized in the University Hospital, and that this group of patients might be in more severe condition than others. In addition, this study was not able to include hidden data indicating the severity of acute aortic dissection, except those contained in the obtained medical records, which may affect patients’ prognoses. Furthermore, confounding by indication cannot be excluded when considering the use of anti-thrombotic drugs in hospitalized patients with type-A aortic dissection. The postoperative application of drugs was based on each patient’s condition, therefore generating an intention-to-treat bias. Importantly, no data were available detailing long-term prognosis, and no data were included regarding antagonists for anti-thrombotic drugs. We were not able to collect all data regarding other cardiovascular outcomes during hospitalization, such as visceral ischemia, kidney ischemia, limb ischemia, blood transfusion, and extent of surgical therapy, because some of these data had not been retained over the long period covered by the study. Moreover, blood pressure and heart rate data were collected at the point when patients were transferred, even if they had experienced cardiogenic shock or atrial fibrillation.

## 5. Conclusions

This study has demonstrated that the use of anti-thrombotic drugs did not worsen in-hospital mortality in patients with acute aortic dissection, suggesting that practitioners should not hesitate to administer anti-thrombotic drugs if indicated.

## Figures and Tables

**Table 1:** Clinical characteristics of TRANSFERRED acute aortic dissection patients with and without anti-thrombotic therapy.

		Stanford Type A (n = 454)			Stanford Type B (n = 231)		
Anti-Thrombotic Therapy		Yes	No	*p* Value	Yes	No	*p* Value
(at the Onset)		(n = 70)	(n = 384)		(n = 38)	(n = 193)	
Hospital stay (days)		38.3 ± 35.6	28.9 ± 22.1	0.04	28.7 ± 28.7	26.1 ± 20.5	0.59
Age (years old)		74.8 ± 8.9	68.2 ± 12.2	<0.01	69.8 ± 15.0	66.0± 11.8	0.14
Sex (male)		42 (60.0%)	183 (47.6%)	0.06	21 (55.3%)	137 (71.0%)	0.06
Surgical/endovascular treatment		59 (84.3%)	314 (81.8%)	0.61	3 (7.9%)	30 (15.5%)	0.22
JCS	0	52 (74.3%)	269 (70.1%)	0.89	37 (97.4%)	180 (93.3%)	0.75
1–3		8 (11.4%)	56 (14.6%)		1 (2.6%)	8 (4.2%)	
10–30		5 (7.1%)	31 (8.1%)		0 (0.0%)	3 (1.6%)	
100–300		5 (7.1%)	28 (7.3%)		0 (0.0%)	2 (1.0%)	
Medical history							
A Fib		22 (31.4%)	13 (3.4%)	<0.01	9 (23.7%)	2 (1.0%)	<0.01
CAD		14 (20.0%)	13 (3.4%)	<0.01	8 (21.1%)	3 (1.6%)	<0.01
Other CVD		27 (38.6%)	36 (9.7%)	<0.01	12 (31.6%)	19 (10.0%)	<0.01
PAD		19 (27.1%)	40 (10.8%)	<0.01	14 (36.8%)	36 (18.7%)	0.01
VTE		1 (1.4%)	1 (0.3%)	0.18	0 (0.0%)	0 (0.0%)	
HT		66 (94.3%)	320 (86.5%)	0.07	37 (97.4%)	181 (94.8%)	0.49
DM		7 (10.0%)	26 (7.1%)	0.39	7 (18.9%)	24 (12.6%)	0.3
DLp		18 (25.7%)	78 (21.1%)	0.4	19 (50.0%)	38 (19.9%)	<0.01
Genetic and others		1 (1.4%)	8 (2.1%)	0.69	4 (10.5%)	4 (2.1%)	<0.01
Smoking		30 (44.8%)	133 (36.7%)	0.21	20 (54.1%)	127 (67.2%)	0.13
Alcohol		28 (41.8%)	109 (30.2%)	0.06	12 (32.4%)	83 (43.9%)	0.2
Surgical history							
post-AVR		6 (8.6%)	2 (0.5%)	<0.01	7 (18.4%)	0 (0.0%)	<0.01
post-MVR		0 (0.0%)	0 (0.0%)		1 (2.6%)	0 (0.0%)	0.02
CABG		2 (2.9%)	1 (0.3%)	0.02	2 (5.3%)	0 (0.0%)	<0.01
Intervention to aortic aneurysm and/or dissection		11 (15.7%)	14 (3.8%)	<0.01	15 (39.5%)	13 (6.8%)	<0.01
Primary outcome							
All-cause death		10 (14.3%)	63 (16.4%)	0.66	1 (2.6%)	14 (7.3%)	0.29
Secondary outcomes							
Major bleeding		5 (7.1%)	25 (7.0%)	0.96	1 (2.6%)	7 (3.6%)	0.75
Infarction by aortic dissection		18 (25.7%)	78 (21.7%)	0.46	0 (0%)	20 (10.4%)	0.04
Paroxysmal A Fib		19 (27.1%)	93 (25.9%)	0.83	2 (5.3%)	8 (4.1%)	0.77
Medication on admission							
RAAS inhibitor		31(45.6%)	93 (29.1%)	<0.01	22 (57.9%)	52 (28.1%)	<0.01
CCB		29 (42.7%)	97 (30.3%)	0.05	16 (42.1%)	51 (27.6%)	0.08
β-blocker		26 (38.2%)	31 (9.7%)	<0.01	12 (31.6%)	29 (15.7%)	0.02
diuretics		9 (13.2%)	18 (5.6%)	0.03	2 (5.3%)	8 (4.3%)	0.8
α-blocker		1 (1.5%)	9 (2.8%)	0.53	0 (0%)	7 (3.8%)	0.22
warfarin		25 (35.7%)	0 (0.0%)		13 (34.2%)	0 (0.0%)	
DOAC		6 (8.6%)	0 (0.0%)		3 (7.9%)	0 (0.0%)	
aspirin		27 (38.6%)	0 (0.0%)		20 (52.6%)	0 (0.0%)	
clopidogrel		12 (17.1%)	0 (0.0%)		5 (13.2%)	0 (0.0%)	
cilostazol		8 (11.4%)	0 (0.0%)		4 (10.5%)	0 (0.0%)	
prasugrel		0 (0.0%)	0 (0.0%)		0 (0.0%)	0 (0.0%)	
Discharge medicine							
RAAS inhibitor		34 (57.6%)	199 (64.0%)	0.35	23 (62.2%)	149 (84.7%)	<0.01
CCB		26 (44.1%)	162 (52.1%)	0.26	28 (75.7%)	163 (92.6%)	<0.01
β-blocker		46 (78.0%)	248 (79.7%)	0.76	33 (89.2%)	158 (89.8%)	0.92
diuretics		21 (35.6%)	103 (33.1%)	0.71	8 (21.6%)	47 (26.7%)	0.91
α-blocker		2 (3.4%)	11 (3.5%)	0.96	2 (5.4%)	14 (8.0%)	0.59
warfarin		17 (28.3%)	59 (18.9%)	0.09	8 (21.6%)	2 (1.1%)	<0.01
DOAC		6 (10.0%)	15 (4.8%)	0.11	3 (8.1%)	2 (1.1%)	0.01
aspirin		24 (40.0%)	76 (24.3%)	0.01	12 (32.4%)	8 (4.5%)	<0.01
clopidogrel		5 (8.3%)	6 (1.9%)	<0.01	5 (13.5%)	0 (0.0%)	<0.01
cilostazol		5 (8.3%)	1 (0.3%)	<0.01	1 (2.7%)	0 (0.0%)	0.03
prasugrel		0 (0.0%)	0 (0.0%)		0 (0.0%)	0 (0.0%)	

Data are mean ± SE, or mean (%), JCS; Japan Coma Scale, A Fib; atrial fibrillation, CAD; coronary artery diseases, CVD; cardiovascular diseases, PAD; peripheral arterial diseases, VTE; venous thromboembolism, LV; left ventricular, HT; hypertension, DM; diabetes mellitus, DLp: dyslipidemia, AVR; aortic valve replacement, MVR; mitral valve replacement, CABG; coronary artery bypass grafting, RAAS; renin-angiotensin-aldosterone system, CCB; calcium channel blocker, DOAC; direct oral anti-coagulant. Genetic and others includes Marfan syndrome, Loeys-Dietz syndrome, and Behçet’s disease.

**Table 2:** Clinical characteristics of TRANSFERRED acute aortic dissection patients with and without anti-coagulant therapy.

Anti-Coagulant Therapy		Stanford Type A (n = 454)			Stanford Type B (n = 231)		
		Yes	No	*p* Value	Yes	No	*p* Value
(at the Onset)		(n = 31)	(n = 423)		(n = 16)	(n = 215)	
Hospital stay (days)		39.1 ± 34.2	29.7 ± 24.0	0.15	37.5 ± 37.8	25.7 ± 20.3	0.23
Age (years old)		75.3 ± 8.9	68.8 ± 12.1	<0.01	61.8 ± 18.3	67.0 ± 11.9	0.28
Sex (male)		20 (64.5%)	205 (48.5%)	0.084	8 (50.0%)	150 (69.8%)	0.1
Surgical/endovascular treatment		28 (90.3%)	345 (81.6%)	0.22	2 (12.5%)	31 (14.4%)	0.83
JCS	0	25 (80.7%)	296 (69.9%)	0.39	15 (93.8%)	202 (94.0%)	0.89
1–3		4 (12.9%)	60 (14.2%)		1 (6.3%)	8 (3.7%)	
10–30		2 (6.5%)	34 (8.0%)		0 (0.0%)	3 (1.4%)	
100–300		0 (0.0%)	33 (7.8%)		0 (0.0%)	2 (0.9%)	
Medical history							
A Fib		18 (58.1%)	17 (4.1%)	<0.01	8 (50.0%)	3 (1.4%)	<0.01
CAD		3 (9.7%)	24 (5.8%)	0.39	1 (6.3%)	10 (4.7%)	0.78
Other CVD		8 (25.8%)	55 (13.4%)	0.056	3 (18.8%)	28 (13.2%)	0.53
PAD		4 (12.9%)	55 (13.4%)	0.94	7 (43.8%)	43 (20.2%)	0.03
VTE		1 (3.2%)	1 (0.2%)	0.017	0 (0.0%)	0 (0.0%)	
HT		29 (93.6%)	357 (87.3%)	0.31	15 (93.8%)	203 (95.3%)	0.78
DM		3 (9.7%)	30 (7.4%)	0.64	4 (25.0%)	27 (12.7%)	0.17
DLp		3 (9.7%)	93 (22.8%)	0.089	6 (37.5%)	51 (23.9%)	0.23
Genetic and others		0 (0.0%)	9 (2.2%)	0.4	4 (25.0%)	4 (1.9%)	<0.01
Smoking		14 (45.2%)	149 (37.4%)	0.39	7 (43.8%)	140 (66.7%)	0.06
Alcohol		14 (45.2%)	123 (31.0%)	0.1	4 (25.0%)	91 (43.3%)	0.15
Surgical history							
post-AVR		5 (16.1%)	3 (0.7%)	<0.01	7 (43.8%)	0 (0.0%)	<0.01
post-MVR		0 (0.0%)	0 (0.0%)		1 (6.3%)	0 (0.0%)	<0.01
CABG		0 (0.0%)	3 (0.7%)	0.63	0 (0.0%)	2 (0.9%)	0.7
Intervention to aortic aneurysm and/or dissection		3 (9.7%)	22 (5.4%)	0.32	7 (43.8%)	21 (9.9%)	<0.01
Primary outcome							
All-cause death		4 (12.9%)	69 (16.3%)	0.62	1 (6.3%)	14 (6.5%)	0.97
Secondary outcomes							
Major bleeding		3 (9.7%)	27 (6.8%)	0.54	1 (6.3%)	7 (3.3%)	0.53
Infarction by aortic dissection		10 (32.3%)	86 (21.6%)	0.17	0 (0.0%)	20 (9.4%)	0.2
Paroxysmal A Fib		7 (22.6%)	105 (26.4%)	0.64	1 (6.3%)	9 (4.2%)	0.7
Medication on admission							
RAAS inhibitor		15 (50.0%)	109 (30.5%)	0.027	6 (37.5%)	68 (32.9%)	0.7
CCB		11 (36.7%)	115 (32.1%)	0.61	5 (31.3%)	62 (30.0%)	0.91
β-blocker		15 (50.0%)	42 (11.7%)	<0.01	9 (56.3%)	32 (15.5%)	<0.01
diuretics		6 (20.0%)	21 (5.9%)	0.004	1 (6.3%)	9 (4.4%)	0.72
α-blocker		0 (0.0%)	10 (2.8%)	0.35	0 (0.0%)	7 (3.4%)	0.45
warfarin		25 (80.7%)	0 (0.0%)	<0.01	13 (81.3%)	0 (0.0%)	<0.01
DOAC		6 (19.4%)	0 (0.0%)	<0.01	3 (18.8%)	0 (0.0%)	<0.01
aspirin		7 (22.6%)	20 (5.6%)	0.0003	5 (31.3%)	15 (7.3%)	0.001
clopidogrel		0 (0.0%)	12 (3.3%)	0.3	0 (0.0%)	5 (2.4%)	0.53
cilostazol		0 (0.0%)	8 (2.2%)	0.4	0 (0.0%)	4 (2.0%)	0.57
prasugrel		0 (0.0%)	0 (0.0%)		0 (0.0%)	0 (0.0%)	
Discharge medicine							
RAAS inhibitor		15 (55.6%)	218 (63.6%)	0.41	10 (66.7%)	162 (81.8%)	0.15
CCB		11 (40.7%)	177 (51.6%)	0.28	12 (80.0%)	179 (90.4%)	0.2
β-blocker		19 (70.4%)	275 (80.2%)	0.22	13 (86.7%)	178 (89.9%)	0.69
diuretics		9 ((33.3%)	115 (33.5%)	0.98	3 (20.0%)	52 (26.3%)	0.59
α-blocker		0 (0.0%)	13 (3.8%)	0.3	0 (0.0%)	16 (8.1%)	0.25
warfarin		14 (51.9%)	62 (17.9%)	<0.01	8 (53.3%)	2 (1.0%)	<0.01
DOAC		6 (22.2%)	15 (4.3%)	<0.01	3 (20.0%)	2 (1.0%)	<0.01
aspirin		11 (40.7%)	89 (25.7%)	0.09	2 (13.3%)	18 (9.1%)	0.58
clopidogrel		0 (0.0%)	11 (3.2%)	0.35	0 (0.0%)	5 (2.5%)	0.53
cilostazol		1 (3.7%)	5 (1.5%)	0.37	0 (0.0%)	1 (0.5%)	0.78
prasugrel		0 (0.0%)	0 (0.0%)		0 (0.0%)	0 (0.0%)	

Data are mean ± SE, or mean (%), JCS; Japan Coma Scale, A Fib; atrial fibrillation, CAD; coronary artery diseases, CVD; cardiovascular diseases, PAD; peripheral arterial diseases, VTE; venous thromboembolism, LV; left ventricular, HT; hypertension, DM; diabetes mellitus, DLp: dyslipidemia, AVR; aortic valve replacement, MVR; mitral valve replacement, CABG; coronary artery bypass grafting, RAAS; renin-angiotensin-aldosterone system, CCB; calcium channel blocker, DOAC; direct oral anti-coagulant. Genetic and others includes Marfan syndrome, Loeys-Dietz syndrome, and Behçet’s disease.

**Table 3:** Clinical characteristics of TRANSFERRED acute aortic dissection patients with and without anti-platelet therapy.

Anti-Platelet Therapy		Stanford Type A (n = 454)			Stanford Type B (n = 231)		
		Yes	No	*p* Value	Yes	No	*p* Value
(at the Onset)		(n = 46)	(n = 408)		(n = 27)	(n = 204)	
Hospital stay (days)		37.2 ± 35.2	29.6 ± 23.3	0.16	22.9 ± 18.2	27.0 ± 22.5	0.37
Age (years old)		74.0 ± 8.5	68.7 ± 12.2	<0.01	72.0 ± 14.9	65.9 ± 12.0	0.02
Sex (male)		27 (58.7%)	198 (48.5%)	0.19	11 (40.7%)	62 (30.4%)	0.28
Surgical/endovascular treatment		37 (80.4%)	336 (82.4%)	0.74	1 (3.7%)	32 (15.7%)	0.09
JCS	0	31 (67.4%)	290 (71.1%)	0.78	27 (100%)	190 (93.1%)	0.58
1–3		6 (13.0%)	58 (14.2%)		0 (0.0%)	9 (4.4%)	
10–30		4 (8.7%)	32 (7.8%)		0 (0.0%)	3 (1.5%)	
100–300		5 (10.9%)	28 (6.9%)		0 (0.0%)	2 (1.0%)	
Medical history							
A Fib		4 (8.7%)	31 (7.8%)	0.83	3 (11.1%)	8 (4.0%)	0.1
CAD		14 (30.4%)	13 (3.3%)	<0.01	7 (25.9%)	4 (2.0%)	<0.01
Other CVD		23 (50.0%)	40 (10.1%)	<0.01	10 (37.0%)	21 (10.4%)	<0.01
PAD		16 (34.8%)	43 (10.9%)	<0.01	9 (33.3%)	41 (20.3%)	0.12
VTE		0 (0.0%)	2 (0.5%)	0.63	0 (0.0%)	0 (0.0%)	
HT		44 (95.7%)	342 (86.8%)	0.08	26 (96.3%)	192 (95.1%)	0.78
DM		4 (8.7%)	29 (7.4%)	0.75	3 (11.5%)	28 (13.9%)	0.75
DLp		16 (34.8%)	80 (20.4%)	0.025	14 (51.9%)	43 (21.3%)	<0.01
Genetic and others		1 (2.2%)	8 (2.0%)	0.95	2 (7.4%)	6 (3.0%)	0.24
Smoking		20 (46.5%)	143 (37.1%)	0.23	15 (57.7%)	132 (66.0%)	0.4
Alcohol		17 (39.5%)	120 (31.2%)	0.26	10 (38.5%)	85 (42.5%)	0.69
Surgical history							
post-AVR		2 (4.4%)	6 (1.5%)	0.18	3 (11.1%)	4 (2.0%)	0.01
post-MVR		0 (0.0%)	0 (0.0%)		0 (0.0%)	1 (0.5%)	0.71
CABG		2 (4.4%)	1 (0.3)	0.001	2 (7.4%)	0 (0.0%)	<0.01
Intervention to aortic aneurysm and/or dissection		9 (19.6%)	16 (4.1%)	<0.01	10 (37.0%)	18 (9.0%)	<0.01
Primary outcome							
All-cause death		8 (17.4%)	65 (15.9%)	0.8	0 (0.0%)	15 (7.4%)	0.15
Secondary outcomes							
Major bleeding		3 (6.5%)	27 (7.1%)	0.89	0 (0.0%)	8 (4.0%)	0.29
Infarction by aortic dissection		12 (26.1%)	84 (21.9%)	0.52	0 (0.0%)	20 (9.9%)	0.087
Paroxysmal A Fib		14 (30.4%)	98 (25.6%)	0.48	1 (3.7%)	9 (4.5%)	0.86
Medication on admission							
RAAS inhibitor		20 (44.4%)	104 (30.3%)	0.056	17 (63.0)	57 (29.1%)	<0.01
CCB		20 (44.4%)	106 (30.9%)	0.068	11 (40.7%)	56 (28.6%)	0.2
β-blocker		15 (33.3%)	42 (12.2%)	<0.01	5 (18.5%)	36 (18.4%)	0.98
diuretics		3 (6.7%)	24 (7.0%)	0.93	1 (3.7%)	9 (4.6%)	0.83
α-blocker		1 (2.2%)	9 (2.6%)	0.87	0 (0.0%)	7 (3.6%)	0.32
warfarin		7 (15.2%)	18 (5.2%)	0.009	3 (11.1%)	10 (5.2%)	0.22
DOAC		0 (0.0%)	6 (1.7%)	0.37	2 (7.4%)	1 (0.5%)	0.004
aspirin		27 (58.7%)	0 (0.0%)	<0.01	20 (74.1%)	0 (0.0%)	<0.01
clopidogrel		12 (26.1%)	0 (0.0%)	<0.01	5 (18.5%)	0 (0.0%)	<0.01
cilostazol		8 (17.4%)	0 (0.0%)	<0.01	4 (14.8%)	0 (0.0%)	<0.01
prasugrel		0 (0.0%)	0 (0.0%)		0 (0.0%)	0 (0.0%)	
Discharge medicine							
RAAS inhibitor		21 (56.8%)	212 (63.7%)	0.41	14 (51.9%)	158 (85.0%)	<0.01
CCB		17 (46.0%)	171 (51.4%)	0.53	19 (70.4%)	172 (92.5%)	<0.01
β-blocker		32 (86.5%)	262 (78.7%)	0.26	23 (85.2%)	168 (90.3%)	0.41
diuretics		13 (35.1%)	111 (33.3%)	0.83	5 (18.5%)	50 (26.9%)	0.35
α-blocker		2 (5.4%)	11 (3.3%)	0.51	2 (7.4%)	14 (7.5%)	0.98
warfarin		6 (15.8%)	70 (20.9%)	0.46	2 (7.4%)	8 (4.3%)	0.47
DOAC		0 (0.0%)	21 (6.3%)	0.11	2 (7.4%)	3 (1.6%)	0.06
aspirin		17 (44.7%)	83 (24.8%)	0.009	11 (40.7%)	9 (4.8%)	<0.01
clopidogrel		5 (13.2%)	6 (1.8%)	<0.01	5 (18.5%)	0 (0.0%)	<0.01
cilostazol		4 (10.5%)	2 (0.6%)	<0.01	1 (3.7%)	0 (0.0%)	0.008
prasugrel		0 (0.0%)	0 (0.0%)		0 (0.0%)	0 (0.0%)	

Data are mean ± SE, or mean (%), JCS; Japan Coma Scale, A Fib; atrial fibrillation, CAD; coronary artery diseases, CVD; cardiovascular diseases, PAD; peripheral arterial diseases, VTE; venous thromboembolism, LV; left ventricular, HT; hypertension, DM; diabetes mellitus, DLp: dyslipidemia, AVR; aortic valve replacement, MVR; mitral valve replacement, CABG; coronary artery bypass grafting, RAAS; renin-angiotensin-aldosterone system, CCB; calcium channel blocker, DOAC; direct oral anti-coagulant. Genetic and others includes Marfan syndrome, Loeys-Dietz syndrome, and Behçet’s disease.

**Table 4:** Cox proportional hazard model for all-cause mortality in TRANSFERRED acute aortic dissection patients.

Type A	β	SE	*p*-Value	Type B	β	SE	*p*-Value
Age	0.01	0.01	0.23	Age	0.0005	0.02	0.98
Sex	−0.02	0.23	0.94	Sex	−0.64	0.65	0.32
Systolic BP	−0.02	<0.01	<0.01	Systolic BP	−0.03	0.01	0.02
Diastolic BP	−0.01	0.008	0.19	Diastolic BP	−0.02	0.02	0.24
Heart rate	0.011	0.006	0.05	Heart rate	0.03	0.01	0.04
eGFR	−0.028	0.006	<0.01	eGFR	−0.02	0.01	0.04
Surgery	−2.39	0.25	<0.01	Surgery	0.77	0.59	0.19
JCS	0.86	0.1	<0.01	JCS	1.23	0.25	<0.01
Past history				Past history			
A Fib	−1.05	0.72	0.15	A Fib	1.93	0.67	<0.01
CAD	0.76	0.4	0.06	CAD	−14.08	1484	0.99
Other CVD	0.005	0.36	0.99	Other CVD	0.18	0.77	0.81
PAD	0.16	0.35	0.64	PAD	0.47	0.6	0.43
VTE	−12.01	744.56	0.99	VTE	0		
LV dysfunction	−13.03	789.73	0.99	LV dysfunction	0		
HT	−0.8	0.31	0.01	HT	14.09	1400	0.99
DM	−0.55	0.59	0.35	DM	0.12	0.77	0.87
DLp	−0.23	0.33	0.49	DLp	−0.04	0.66	0.95
Genetic and others	0.57	0.62	0.35	Genetic and others	−14.07	1509	0.99
Smoking	−0.64	0.31	0.04	Smoking	0.21	0.6	0.72
Alcohol	−0.68	0.34	0.04	Alcohol	−0.2	0.57	0.73
post-AVR	1.02	0.59	0.09	post-AVR	−14.09	1577	0.99
post-MVR	0			post-MVR	−11.01	1501	0.99
CABG	−12.01	627.8	0.98	CABG	−12.01	1543	0.99
Intervention to aortic aneurysm and/or dissection	0.27	0.47	0.57	Intervention to aortic aneurysm and/or dissection	−0.45	1.04	0.67
Complication				Complication			
Major bleeding	1.52	0.32	<0.01	Major bleeding	3.87	0.59	<0.01
Infarction by aortic dissection	1.54	0.27	<0.01	Infarction by aortic dissection	2.04	0.57	<0.01
Paroxysmal A Fib	−1.66	0.52	<0.01	Paroxysmal A Fib	−14.09	1435	0.99
Medication on admission				Medication on admission			
RAAS inhibitor	−0.06	0.31	0.85	RAAS inhibitor	−16.67	1711	0.99
CCB	0.36	0.29	0.22	CCB	0.14	0.61	0.82
β-blocker	0.04	0.39	0.93	β-blocker	−0.2	0.78	0.8
diuretics	0.6	0.44	0.17	diuretics	1.9	0.79	0.02
α-blocker	1.2	0.52	0.02	α-blocker	−14.05	1838	0.99
warfarin	0.16	0.53	0.77	warfarin	0.4	1.06	0.71
DOAC	−13.03	787.37	0.99	DOAC	−13.02	2047	0.99
aspirin	0.8	0.41	0.05	aspirin	−15.16	1903	0.99
clopidogrel	−14.17	771.42	0.99	clopidogrel	−13.04	2043	0.99
cilostazol	−13.03	673.45	0.98	cilostazol	−13.05	1571	0.99
prasugrel	0			prasugrel	0		
other anti-platelet drug	1.18	1.01	0.24	other anti-platelet drug	−12.01	2086	0.99
Anti-coagulant (warfarin or DOAC)	−0.33	0.52	0.52	Anti-coagulant (warfarin or DOAC)	−0.12	1.04	0.91
Anti-platelet drug (aspirin, clopidogrel, cilostazol, or prasugrel)	−0.02	0.38	0.96	Anti-platelet drug (aspirin, clopidogrel, cilostazol, or prasugrel)	−15.19	1457	0.99
Both anti-coagulant and anti-platelet	0.55	0.72	0.44	Both anti-coagulant and anti-platelet	−13.03	1218	0.99
Anti-coagulant or anti-platelet	−0.26	0.34	0.45	Anti-coagulant or anti-platelet	−1.02	1.04	0.33

SE; standard error, HR, hazard ratio, BP; blood pressure, eGFR; estimated glomerular filtration rate, JCS; Japan Coma Scale, A Fib; atrial fibrillation, CAD; coronary artery diseases, CVD; cardiovascular diseases, PAD; peripheral arterial diseases, VTE; venous thromboembolism, LV; left ventricular, HT; hypertension, DM; diabetes mellitus, DLp: dyslipidemia, AVR; aortic valve replacement, MVR; mitral valve replacement, CABG; coronary artery bypass grafting, RAAS; renin-angiotensin-aldosterone system, CCB; calcium channel blocker, DOAC; direct oral anti-coagulant. Genetic and others includes Marfan syndrome, Loeys-Dietz syndrome, and Behçet’s disease.

**Table 5:** Age and sex-adjusted Cox proportional hazard model for all-cause mortality in TRANSFERRED acute aortic dissection patients.

Type A (Age and Sex-Adjusted)	β	SE	*p*-Value	Type B (Age and Sex-Adjusted)	β	SE	*p*-Value
Systolic BP	−0.02	0.004	<0.01	Systolic BP	−0.03	0.01	0.01
Diastolic BP	−0.01	0.008	0.17	Diastolic BP	−0.02	0.02	0.18
Heart rate	0.01	0.006	0.07	Heart rate	0.03	0.02	0.05
eGFR	−0.03	0.006	<0.01	eGFR	−0.02	0.01	0.02
Surgery	−2.38	0.25	<0.01	Surgery	0.85	0.59	0.15
JCS	0.85	0.1	<0.01	JCS	1.23	0.26	<0.01
Past history				Past history			
A Fib	−1.21	0.72	0.1	A Fib	2.05	0.69	<0.01
CAD	0.7	0.4	0.09	CAD	−14	1480	0.99
Other CVD	−0.08	0.37	0.83	Other CVD	0.16	0.78	0.83
PAD	0.05	0.36	0.89	PAD	0.5	0.61	0.41
VTE	−12.06	752.05	0.99	VTE	0		
LV dysfunction	−12.99	788.9	0.99	LV dysfunction	0		
HT	−0.88	0.32	<0.01	HT	14.07	1400	0.99
DM	−0.58	0.59	0.33	DM	0.17	0.77	0.82
DLp	−0.2	0.34	0.56	DLp	−0.02	0.66	0.98
Genetic and others	0.71	0.63	0.26	Genetic and others	−14.11	1513	0.99
Smoking	−0.86	0.36	0.02	Smoking	−0.01	0.7	0.99
Alcohol	−0.8	0.38	0.03	Alcohol	−0.39	0.6	0.52
post-AVR	1.01	0.61	0.1	post-AVR	−14.02	1587	0.99
post-MVR	0			post-MVR	−11.13	1515	0.99
CABG	−12.24	633.99	0.98	CABG	−12.16	1558	0.99
Intervention to aortic aneurysm and/or dissection	0.18	0.49	0.72	Intervention to aortic aneurysm and/or dissection	−0.41	1.05	0.7
Complication				Complication			
Major bleeding	1.48	0.32	<0.01	Major bleeding	4.24	0.71	<0.01
Infarction by aortic dissection	1.52	0.27	<0.01	Infarction by aortic dissection	2.04	0.57	<0.01
Paroxysmal A Fib	−1.75	0.52	<0.01	Paroxysmal A Fib	−13.95	1435	0.99
Medication on admission				Medication on admission			
RAAS inhibitor	−0.14	0.31	0.66	RAAS inhibitor	−16.6	1710	0.99
CCB	0.29	0.29	0.33	CCB	0.33	0.63	0.6
β-blocker	−0.05	0.39	0.89	β-blocker	−0.05	0.78	0.94
diuretics	0.48	0.44	0.27	diuretics	1.83	0.79	0.02
α-blocker	1.16	0.52	0.03	α-blocker	−13.97	1835	0.99
warfarin	−0.002	0.54	0.99	warfarin	0.7	1.07	0.51
DOAC	−13.24	796.32	0.99	DOAC	−13.25	2020	0.99
aspirin	0.75	0.42	0.07	aspirin	−15.33	1875	0.99
clopidogrel	−14.35	776.75	0.99	clopidogrel	−13.26	2052	0.99
cilostazol	−13.12	675.96	0.98	cilostazol	−13.68	1616	0.99
prasugrel	0			prasugrel	0		
other anti-platelet drug	1.14	1.02	0.27	other anti-platelet drug	−12.41	2152	0.99
Anti-coagulant (warfarin or DOAC)	−0.48	0.53	0.36	Anti-coagulant (warfarin or DOAC)	0.05	1.05	0.96
Anti-platelet drug (aspirin, clopidogrel, cilostazol, or prasugrel)	−0.11	0.38	0.78	Anti-platelet drug (aspirin, clopidogrel, cilostazol, or prasugrel)	−15.19	1449	0.99
Both anti-coagulant and anti-platelet	0.49	0.72	0.5	Both anti-coagulant and anti-platelet	−13.01	1217	0.99
Anti-coagulant or anti-platelet	−0.41	0.36	0.25	Anti-coagulant or anti-platelet	−0.94	1.05	0.37

SE; standard error, HR, hazard ratio, BP; blood pressure, eGFR; estimated glomerular filtration rate, JCS; Japan Coma Scale, A Fib; atrial fibrillation, CAD; coronary artery diseases, CVD; cardiovascular diseases, PAD; peripheral arterial diseases, VTE; venous thromboembolism, LV; left ventricular, HT; hypertension, DM; diabetes mellitus, DLp: dyslipidemia, AVR; aortic valve replacement, MVR; mitral valve replacement, CABG; coronary artery bypass grafting, RAAS; renin-angiotensin-aldosterone system, CCB; calcium channel blocker, DOAC; direct oral anti-coagulant. Genetic and others includes Marfan syndrome, Loeys-Dietz syndrome, and Behçet’s disease.

**Table 6:** Clinical characteristics of HOSPITALIZED acute aortic dissection with and without anti-thrombotic therapy.

		Stanford Type A (n = 424)			Stanford Type B (n = 226)		
Anti-Thrombotic Therapy		Yes	No	*p* Value	Yes	No	*p* Value
(During Hospitalization)		(n = 182)	(n = 242)		(n = 42)	(n = 184)	
Hospital stay (days)		36.9 ± 24.5	29.1± 23.8	<0.01	35.6 ± 30.8	25.7 ±20.0	0.01
Age (years old)		69.9 ± 11.3	68.3 ± 12.3	0.17	67.8 ± 15.0	66.4 ± 11.8	0.51
Sex (male)		91 (50.0%)	120 (49.6%)	0.9	23 (54.8%)	131 (71.2%)	0.04
Surgical/endovascular treatment		177 (97.3%)	194 (80.2%)	<0.01	6 (14.3)	25 (13.6)	0.91
JCS	0	140 (76.9%)	173 (71.5%)	0.52	41 (97.6%)	174 (94.6%)	0.82
1–3		24 (13.2%)	34 (14.1%)		1 (2.4%)	7 (3.8%)	
10–30		12 (6.6%)	22 (9.1%)		0 (0.0%)	2 (1.1%)	
100–300		6 (3.3%)	13 (5.4%)		0 (0.0%)	1 (0.5%)	
Medical history							
A Fib		25 (13.7%)	9 (3.7%)	<0.01	6 (14.3%)	4 (2.2%)	<0.01
CAD		13 (7.1%)	11 (4.6%)	0.27	7 (16.7%)	4 (2.2%)	<0.01
Other CVD		34 (18.7%)	27 (11.3%)	0.03	9 (21.4%)	20 (10.9%)	0.06
PAD		23 (12.6%)	32 (13.5%)	0.81	12 (28.6%)	37 (20.1%)	0.49
VTE		2 (1.1%)	0 (0.0%)	0.11	0 (0.0%)	0 (0.0%)	
HT		159 (87.4%)	213 (90.3%)	0.35	40 (95.2%)	175 (95.1%)	0.97
DM		17 (9.3%)	15 (6.4%)	0.26	7 (17.1%)	23 (12.5%)	0.44
DLp		37 (20.3%)	55 (23.4%)	0.45	16 (38.1%)	39 (21.2%)	0.02
Genetic and others		7 (3.8%)	1 (0.4%)	0.01	4 (9.5%)	4 (2.2%)	0.02
Smoking		65 (35.7%)	95 (41.1%)	0.26	23 (56.1%)	122 (67.0%)	0.18
Alcohol		60 (33.0%)	75 (32.6%)	0.94	13 (31.7%)	81 (44.5%)	0.13
Surgical history							
post-AVR		5 (2.7%)	2 (0.8%)	0.13	6 (14.3%)	1 (0.5%)	<0.01
post-MVR		0 (0.0%)	0 (0.0%)		0 (0.0%)	1 (0.5%)	0.63
CABG		2 (1.1%)	1 (0.4%)	0.42	2 (4.8%)	0 (0.0%)	<0.01
Intervention to aortic aneurysm and/or dissection		13 (7.1%)	10 (4.2%)	0.2	10 (24.4%)	17 (9.2%)	<0.01
Primary outcome							
All-cause death		4 (2.2%)	39 (16.1%)	<0.001	1 (2.4%)	9 (4.9%)	0.48
Secondary outcomes							
Major bleeding		8 (4.5%)	18 (7.8%)	0.17	0 (0%)	6 (3.3%)	0.24
Infarction by aortic dissection		45 (25.1%)	41 (17.8%)	0.07	4 (9.5%)	16 (8.7%)	0.86
Paroxysmal A Fib		77 (43.0%)	35 (15.2%)	<0.01	3 (7.1%)	7 (3.8%)	0.34
Medication on admission							
RAAS inhibitor		58 (35.4%)	61 (29.3%)	0.22	18 (42.9%)	56 (31.3%)	0.15
CCB		55 (33.5%)	66 (31.7%)	0.71	12 (28.6%)	54 (30.2%)	0.84
β-blocker		30 (18.3%)	24 (11.5%)	0.07	8 (19.0%)	32 (17.9%)	0.86
diuretics		15 (9.2%)	10 (4.8%)	0.09	1 (2.4%)	7 (3.9%)	0.63
α-blocker		2 (1.2%)	7 (3.4%)	0.18	0 (0%)	7 (3.9%)	0.19
warfarin		20 (12.1%)	4 (1.9%)	<0.01	10(23.8%)	2 (1.1%)	<0.01
DOAC		5 (3.0%)	1 (0.5%)	0.05	3 (7.1%)	0 (0.0%)	<0.01
aspirin		15 (9.0%)	9 (4.3%)	0.06	15 (35.7%)	5 (2.8%)	<0.01
clopidogrel		9 (5.4%)	3 (1.4%)	0.03	5 (11.9%)	0 (0%)	<0.01
cilostazol		4 (2.4%)	4 (1.9%)	0.74	3 (7.1%)	1 (0.5%)	<0.01
prasugrel		0 (0.0%)	0 (0.0%)		0 (0.0%)	0 (0.0%)	
Discharge medicine							
RAAS inhibitor		107 (61.5%)	126 (64.3%)	0.58	28 (68.3%)	144 (83.7%)	0.02
CCB		75 (43.1%)	113 (57.7%)	<0.01	31 (75.6%)	160 (93.0%)	<0.01
β-blocker		146 (83.9%)	148 (75.5%)	0.046	35 (85.4%)	156 (90.7%)	0.31
diuretics		54 (31.0%)	70 (35.7%)	0.34	11 (26.8%)	44 (25.6%)	0.87
α-blocker		5 (2.9%)	8 (4.1%)	0.53	3 (7.3%)	13 (7.6%)	0.96
warfarin		76 (42.7%)	0 (0.0%)	<0.01	10 (24.4%)	0 (0.0%)	<0.01
DOAC		21 (11.8%)	0 (0.0%)	<0.01	5 (12.2%)	0 (0.0%)	<0.01
aspirin		100 (56.2%)	0 (0.0%)	<0.01	20 (48.8%)	0 (0.0%)	<0.01
clopidogrel		11 (6.2%)	0 (0.0%)	<0.01	5 (12.2%)	0 (0.0%)	<0.01
cilostazol		6 (3.4%)	0 (0.0%)	<0.01	1 (2.4%)	0 (0.0%)	0.04
prasugrel		0 (0.0%)	0 (0.0%)		0 (0.0%)	0 (0.0%)	

Data are mean ± SE, or mean (%), JCS; Japan Coma Scale, A Fib; atrial fibrillation, CAD; coronary artery diseases, CVD; cardiovascular diseases, PAD; peripheral arterial diseases, VTE; venous thromboembolism, LV; left ventricular, HT; hypertension, DM; diabetes mellitus, DLp: dyslipidemia, AVR; aortic valve replacement, MVR; mitral valve replacement, CABG; coronary artery bypass grafting, RAAS; renin-angiotensin-aldosterone system, CCB; calcium channel blocker, DOAC; direct oral anti-coagulant. Genetic and others includes Marfan syndrome, Loeys-Dietz syndrome, and Behçet’s disease.

**Table 7:** Clinical characteristics of HOSPITALIZED acute aortic dissection with and without anti-coagulant therapy.

Anti-Coagulant Therapy		Stanford Type A (n = 424)			Stanford Type B (n = 226)		
		Yes	No	*p* Value	Yes	No	*p* Value
(During Hospitalization)		(n = 109)	(n = 315)		(n = 16)	(n = 210)	
Hospital stay (days)		38.3 ± 23.9	30.4 ± 24.3	0.004	36.5 ± 37.8	26.3 ± 20.2	0.3
Age (years old)		70.3 ± 11.3	68.6 ± 12.1	0.2	66.1 ± 18.4	66.7 ± 12.0	0.91
Sex (male)		50 (45.9%)	161 (51.1%)	0.35	9 (56.3%)	145 (69.1%)	0.29
Surgical/endovascular treatment		104 (95.4%)	267 (84.8%)	0.004	2 (12.5%)	29 (13.8%)	0.88
JCS	0	85 (78.0%)	228 (72.4%)	0.61	15 (93.8%)	200 (95.2%)	0.9
1–3		13 (11.9%)	45 (14.3%)		1 (6.3%)	7 (3.3%)	
10–30		6 (5.5%)	28 (8.9%)		0 (0.0%)	2 (1.0%)	
100–300		5 (4.6%)	14 (4.4%)		0 (0.0%)	1 (0.5%)	
Medical history							
A Fib		23 (21.1%)	11 (3.5%)	<0.01	6 (37.5%)	4 (1.9%)	<0.01
CAD		7 (6.4%)	17 (5.5%)	0.71	0 (0.0%)	11 (5.2%)	0.35
Other CVD		19 (17.4%)	42 (13.5%)	0.31	1 (6.3%)	28 (13.3%)	0.41
PAD		13 (11.9%)	42 (13.5%)	0.67	6 (37.5%)	43 (20.5%)	0.11
VTE		2 (1.8%)	0 (0.0%)	0.02	0 (0.0%)	0 (0.0%)	
HT		98 (89.9%)	274 (88.7%)	0.72	15 (93.8%)	200 (95.2%)	0.79
DM		12 (11.0%)	20 (6.5%)	0.13	3 (18.8%)	27 (12.9%)	0.51
DLp		24 (22.0%)	68 (22.1%)	0.99	5 (31.3%)	50 (23.8%)	0.5
Genetic and others		7 (6.4%)	1 (0.3%)	<0.01	3 (18.8%)	5 (2.4%)	<0.01
Smoking		33 (30.3%)	127 (41.8%)	0.03	7 (43.8%)	138 (66.7%)	0.06
Alcohol		30 (27.5%)	105 (34.6%)	0.17	6 (37.5%)	88 (42.5%)	0.7
Surgical history							
post-AVR		3 (2.8%)	4 (1.3%)	0.31	5 (31.3%)	2 (1.0%)	<0.01
post-MVR		0 (0.0%)	0 (0.0%)		0 (0.0%)	1 (0.5%)	0.78
CABG		0 (0.0%)	3 (1.0%)	0.3	0 (0.0%)	2 (1.0%)	0.7
Intervention to aortic aneurysm and/or dissection		7 (6.4%)	16 (5.2%)	0.62	3 (18.8%)	24 (11.5%)	0.39
Primary outcome							
All-cause death		3 (2.8%)	40 (12.7%)	0.003	0 (0.0%)	10 (4.8%)	0.37
Secondary outcomes							
Major bleeding		6 (5.7%)	20 (6.6%)	0.74	0 (0.0%)	6 (2.9%)	0.49
Infarction by aortic dissection		24 (22.6%)	62 (20.4%)	0.62	0 (0.0%)	20 (9.5%)	0.2
Paroxysmal A Fib		59 (55.7%)	53 (17.4%)	<0.01	3 (18.8%)	7 (3.3%)	0.004
Medication on admission							
RAAS inhibitor		36 (37.1%)	83 (30.2%)	0.21	5 (31.3%)	69 (33.7%)	0.84
CCB		38 (39.2%)	83 (30.2%)	0.1	4 (25.0%)	62 (30.2%)	0.66
β-blocker		20 (20.6%)	34 (12.4%)	0.047	6 (37.5%)	34 (16.6%)	0.04
diuretics		12 (12.4%)	13 (4.7%)	0.01	0 (0.0%)	8 (3.9%)	0.42
α-blocker		1 (1.0%)	8 (2.9%)	0.3	0 (0.0%)	7 (3.4%)	0.45
warfarin		17 (17.2%)	7 (2.5%)	<0.01	8 (50.0%)	4 (2.0%)	<0.01
DOAC		5 (5.1%)	1 (0.4%)	0.001	3 (18.8%)	0 (0.0%)	<0.01
aspirin		7 (7.1%)	17 (6.2%)	0.75	4 (25.0%)	16 (7.9%)	0.02
clopidogrel		1 (1.0%)	11 (4.0%)	0.15	0 (0.0%)	5 (2.5%)	0.53
cilostazol		1 (1.0%)	7 (2.5%)	0.37	0 (0.0%)	4 (2.0%)	0.57
prasugrel		0 (0.0%)	0 (0.0%)		0 (0.0%)	0 (0.0%)	
Discharge medicine							
RAAS inhibitor		60 (58.8%)	173 (64.6%)	0.31	11 (68.8%)	161 (81.7%)	0.21
CCB		44 (43.1%)	144 (53.7%)	0.07	13 (81.3%)	178 (90.4%)	0.25
β-blocker		86 (84.3%)	208 (77.6%)	0.15	14 (87.5%)	177 (89.9%)	0.77
diuretics		35 (34.3%)	89 (33.2%)	0.84	3 (18.8%)	52 (26.4%)	0.5
α-blocker		2 (2.0%)	11 (4.1%)	0.32	1 (6.3%)	15 (7.6%)	0.84
warfarin		76 (71.7%)	0 (0.0%)	<0.01	10 (62.5%)	0 (0.0%)	<0.01
DOAC		21 (19.8%)	0 (0.0%)	<0.01	5 (31.3%)	0 (0.0%)	<0.01
aspirin		41 (38.7%)	59 (22.1%)	0.001	1 (6.3%)	19 (9.6%)	0.66
clopidogrel		2 (1.9%)	9 (3.4%)	0.44	0 (0.0%)	5 (2.5%)	0.52
cilostazol		2 (1.9%)	4 (1.5%)	0.79	0 (0.0%)	1 (0.5%)	0.78
prasugrel		0 (0.0%)	0 (0.0%)		0 (0.0%)	0 (0.0%)	

Data are mean ± SE, or mean (%), JCS; Japan Coma Scale, A Fib; atrial fibrillation, CAD; coronary artery diseases, CVD; cardiovascular diseases, PAD; peripheral arterial diseases, VTE; venous thromboembolism, LV; left ventricular, HT; hypertension, DM; diabetes mellitus, DLp: dyslipidemia, AVR; aortic valve replacement, MVR; mitral valve replacement, CABG; coronary artery bypass grafting, RAAS; renin-angiotensin-aldosterone system, CCB; calcium channel blocker, DOAC; direct oral anti-coagulant. Genetic and others includes Marfan syndrome, Loeys-Dietz syndrome, and Behçet’s disease.

**Table 8:** Clinical characteristics of HOSPITALIZED acute aortic dissection with and without anti-platelet therapy.

Anti-Platelet Therapy		Stanford Type A (n = 424)			Stanford Type B (n = 226)		
		Yes	No	*p* Value	Yes	No	*p* Value
(During Hospitalization)		(n = 118)	(n = 306)		(n = 27)	(n = 199)	
Hospital stay (days)		36.3 ± 23.6	31.0 ± 24.6	0.04	31.5 ± 21.5	26.5 ± 22.0	0.26
Age (years old)		68.3 ± 12.2	69.3 ± 11.8	0.4	69.4 ± 12.8	66.3 ± 12.4	0.23
Sex (male)		53 (44.9%)	160 (52.3%)	0.17	12 (44.4%)	60 (30.2%)	0.13
Surgical/endovascular treatment		117 (99.2%)	254 (83.0%)	<0.01	4 (14.8%)	27 (13.6%)	0.86
JCS	0	92 (77.9%)	221 (72.2%)	0.54	27 (100.0%)	188 (94.5%)	0.67
1–3		15 (12.7%)	43 (14.1%)		0 (0.0%)	8 (4.0%)	
10–30		8 (6.8%)	26 (8.5%)		0 (0.0%)	2 (1.0%)	
100–300		3 (2.5%)	16 (5.2%)		0 (0.0%)	1 (0.5%)	
Medical history							
A Fib		7 (5.9%)	27 (8.9%)	0.31	0 (0.0%)	10 (5.0%)	0.23
CAD		11 (9.3%)	13 (4.3%)	0.046	7 (25.9%)	4 (2.0%)	<0.01
Other CVD		24 (20.3%)	37 (12.2%)	0.03	8 (29.6%)	21 (10.6%)	0.005
PAD		14 (11.9%)	41 (13.6%)	0.64	7 (25.9%)	42 (21.1%)	0.57
VTE		1 (0.9%)	1 (0.3%)	0.49	0 (0.0%)	0 (0.0%)	
HT		96 (81.4%)	276 (92.0%)	0.002	26 (96.3%)	189 (95.0%)	0.76
DM		8 (6.8%)	24 (8.0%)	0.67	4 (15.4%)	26 (13.1%)	0.74
DLp		24 (20.3%)	68 (22.7%)	0.59	11 (40.7%)	44 (22.1%)	0.03
Genetic and others		5 (4.2%)	3 (1.0%)	0.03	1 (3.7%)	7 (3.5%)	0.96
Smoking		49 (41.5%)	111 (37.6%)	0.46	17 (65.4%)	128 (65.0%)	0.97
Alcohol		45 (38.1%)	90 (30.6%)	0.14	8 (30.8%)	86 (43.7%)	0.21
Surgical history							
post-AVR		3 (2.5%)	4 (1.3%)	0.39	1 (3.7%)	6 (3.0%)	0.85
post-MVR		0 (0.0%)	0 (0.0%)		0 (0.0%)	1 (0.5%)	0.71
CABG		2 (1.7%)	1 (0.3%)	0.14	2 (7.4%)	0 (0.0%)	<0.01
Intervention to aortic aneurysm and/or dissection		10 (8.5%)	13 (4.3%)	0.09	7 (26.9%)	20 (10.1%)	0.01
Primary outcome							
All-cause death		1 (0.9%)	42 (13.7%)	<0.01	1 (3.7%)	9 (4.5%)	0.85
Secondary outcomes							
Major bleeding		5 (4.3%)	21 (7.2%)	0.28	0 (0.0%)	6 (3.0%)	0.36
Infarction by aortic dissection		31 (26.5%)	55 (18.8%)	0.08	4 (14.8%)	16 (8.0%)	0.24
Paroxysmal A Fib		36 (30.8%)	76 (25.9%)	0.32	0 (0,0%)	10 (5.0%)	0.23
Medication on admission							
RAAS inhibitor		36 (33.6%)	83 (31.3%)	0.66	13 (48.2%)	61 (31.4%)	0.08
CCB		30 (28.0%)	91 (34.3%)	0.24	8 (29.6%)	58 (29.9%)	0.98
β-blocker		18 (16.8%)	36 (13.6%)	0.42	2 (7.4%)	38 (19.6%)	0.12
diuretics		8 (7.6%)	17 (6.4%)	0.69	1 (3.7%)	7 (3.6%)	0.98
α-blocker		1 (0.9%)	8 (3.0%)	0.24	0 (0.0%)	7 (3.6%)	0.32
warfarin		13 (12.0%)	11 (4.1%)	0.005	2 (7.4%)	10 (5.2%)	0.64
DOAC		0 (0.0%)	6 (2.3%)	0.12	0 (0.0%)	3 (1.6%)	0.51
aspirin		14 (13.0%)	10 (3.8%)	0.001	11 (40.7%)	9 (4.7%)	<0.01
clopidogrel		9 (8.3%)	3 (1.1%)	<0.01	5 (18.5%)	0 (0.0%)	<0.01
cilostazol		4 (3.7%)	4 (1.5%)	0.18	3 (11.1%)	1 (0.5%)	<0.01
prasugrel		0 (0.0%)	0 (0.0%)		0 (0.0%)	0 (0.0%)	
Discharge medicine							
RAAS inhibitor		74 (64.9%)	159 (62.1%)	0.61	18 (69.2%)	154 (82.4%)	0.11
CCB		52 (45.6%)	136 (53.1%)	0.18	19 (73.1%)	172 (92.0%)	0.003
β-blocker		96 (84.2%)	198 (77.3%)	0.13	22 (84.6%)	169 (90.4%)	0.37
diuretics		34 (29.8%)	90 (35.2%)	0.32	8 (30.8%)	47 (25.1%)	0.54
α-blocker		4 (3.5%)	9 (3.5%)	0.99	2 (7.7%)	14 (7.5%)	0.97
warfarin		34 (29.1%)	42 (16.4%)	0.005	0 (0.0%)	10 (5.3%)	0.23
DOAC		4 (3.4%)	17 (6.6%)	0.21	0 (0.0%)	5 (2.7%)	0.4
aspirin		99 (84.6%)	1 (0.4%)	<0.01	20 (76.9%)	0 (0.0%)	<0.01
clopidogrel		11 (9.4%)	0 (0.0%)	<0.01	5 (19.2%)	0 (0.0%)	<0.01
cilostazol		6 (5.1%)	0 (0.0%)	<0.01	1 (3.9%)	0 (0.0%)	0.007
prasugrel		0 (0.0%)	0 (0.0%)		0 (0.0%)	0 (0.0%)	

Data are mean ± SE, or mean (%), JCS; Japan Coma Scale, A Fib; atrial fibrillation, CAD; coronary artery diseases, CVD; cardiovascular diseases, PAD; peripheral arterial diseases, VTE; venous thromboembolism, LV; left ventricular, HT; hypertension, DM; diabetes mellitus, DLp: dyslipidemia, AVR; aortic valve replacement, MVR; mitral valve replacement, CABG; coronary artery bypass grafting, RAAS; renin-angiotensin-aldosterone system, CCB; calcium channel blocker, DOAC; direct oral anti-coagulant. Genetic and others includes Marfan syndrome, Loeys-Dietz syndrome, and Behçet’s disease.

**Table 9:** Cox proportional hazard model for all-cause mortality in HOSPITALIZED acute aortic dissection patients.

Type A	β	SE	*p*-Value	Type B	β	SE	*p*-Value
Age	0.006	0.01	0.65	Age	0.004	0.025	0.88
Sex	−0.11	0.31	0.72	Sex	−0.65	0.79	0.41
Systolic BP	−0.02	0.006	<0.01	Systolic BP	−0.011	0.014	0.4
Diastolic BP	−0.005	0.01	0.63	Diastolic BP	−0.002	0.018	0.9
Heart rate	0.01	0.008	0.11	Heart rate	0.033	0.016	0.04
eGFR	−0.028	0.007	<0.01	eGFR	−0.022	0.012	0.08
Surgery	−1.7	0.33	<0.01	Surgery	0.38	0.8	0.63
JCS	0.82	0.13	<0.01	JCS	1.04	0.42	0.01
Past history				Past history			
A Fib	−1.34	1.01	0.19	A Fib	1.79	0.8	0.03
CAD	0.67	0.53	0.21	CAD	−14.08	1721	0.99
Other CVD	0.22	0.42	0.6	Other CVD	−15.23	1650	0.99
PAD	0.04	0.45	0.93	PAD	0.44	0.69	0.53
VTE	−12.01	915.23	0.99	VTE	0		
LV dysfunction	−13.04	930.19	0.99	LV dysfunction	0		
HT	−0.28	0.48	0.56	HT	14.1	1594	0.99
DM	−0.56	0.73	0.44	DM	−0.38	1.06	0.72
DLp	−0.23	0.42	0.58	DLp	−1.02	1.05	0.33
Genetic and others	0.47	0.77	0.54	Genetic and others	−14.07	1681	0.99
Smoking	−0.52	0.36	0.15	Smoking	0.26	0.69	0.71
Alcohol	−0.52	0.38	0.18	Alcohol	−0.15	0.65	0.82
post-AVR	1.03	0.73	0.16	post-AVR	−14.1	1749	0.99
post-MVR	0			post-MVR	−11.01	2104	0.99
CABG	−12.01	776.4	0.99	CABG	−12.01	1962	0.99
Intervention to aortic aneurysm and/or dissection	0.1	0.61	0.87	Intervention to aortic aneurysm and/or dissection	−15.21	1776	0.99
Complication				Complication			
Major bleeding	1.66	0.38	<0.01	Major bleeding	3.82	0.68	<0.01
Infarction by aortic dissection	1.63	0.33	<0.01	Infarction by aortic dissection	2.58	0.66	<0.01
Paroxysmal A Fib	−1.27	0.53	0.02	Paroxysmal A Fib	−14.1	1623	0.99
Medication on admission				Medication on admission			
RAAS inhibitor	−0.073	0.38	0.85	RAAS inhibitor	−16.67	1876	0.99
CCB	0.54	0.35	0.12	CCB	−0.014	0.69	0.98
β-blocker	−0.1	0.49	0.84	β-blocker	−0.81	1.06	0.44
diuretics	0.58	0.54	0.28	diuretics	−14.05	2135	0.99
α-blocker	1.32	0.61	0.03	α-blocker	−14.05	2021	0.99
warfarin	0.23	0.61	0.71	warfarin	−15.13	2435	0.99
DOAC	−13.03	969.9	0.99	DOAC	−13.02	2372	0.99
aspirin	0.61	0.54	0.26	aspirin	−15.17	2114	0.99
clopidogrel	−14.21	884.34	0.99	clopidogrel	−12.03	1628	0.99
cilostazol	−13.04	823.72	0.99	cilostazol	−13.06	1757	0.99
prasugrel	0			prasugrel	0		
other anti-platelet drug	1.69	1.02	0.1	other anti-platelet drug	−12.01	2395	0.99
Anti-thrombus during hospitalization				Anti-thrombus during hospitalization			
Anti-coagulant (warfarin or DOAC)	−1.62	0.6	<0.01	Anti-coagulant (warfarin or DOAC)	−15.13	2087	0.99
Anti-platelet drug (aspirin, clopidogrel, cilostazol, or prasugrel)	−2.88	1.01	<0.01	Anti-platelet drug (aspirin, clopidogrel, cilostazol, or prasugrel)	−0.38	1.06	0.72
Both anti-coagulant and anti-platelet	−15.2	860.56	0.99	Both anti-coagulant and anti-platelet	−12.01	1770	0.99
Anti-coagulant or anti-platelet	−2.12	0.53	<0.01	Anti-coagulant or anti-platelet	−0.91	1.06	0.39

SE; standard error, HR, hazard ratio, BP; blood pressure, eGFR; estimated glomerular filtration rate, JCS; Japan Coma Scale, A Fib; atrial fibrillation, CAD; coronary artery diseases, CVD; cardiovascular diseases, PAD; peripheral arterial diseases, VTE; venous thromboembolism, LV; left ventricular, HT; hypertension, DM; diabetes mellitus, DLp: dyslipidemia, AVR; aortic valve replacement, MVR; mitral valve replacement, CABG; coronary artery bypass grafting, RAAS; renin-angiotensin-aldosterone system, CCB; calcium channel blocker, DOAC; direct oral anti-coagulant. Genetic and others includes Marfan syndrome, Loeys-Dietz syndrome, and Behçet’s disease.

**Table 10:** Age and sex-adjusted Cox proportional hazard model for all-cause mortality in HOSPITALIZED acute aortic dissection patients.

Type A (Age and Sex-Adjusted)	β	SE	*p*-Value	Type B (Age and Sex-Adjusted)	β	SE	*p*-Value
Systolic BP	−0.02	0.006	<0.01	Systolic BP	−0.012	0.014	0.37
Diastolic BP	−0.005	0.01	0.61	Diastolic BP	−0.005	0.019	0.79
Heart rate	0.012	0.008	0.12	Heart rate	0.035	0.018	0.05
eGFR	−0.028	0.007	<0.01	eGFR	−0.025	0.013	0.05
Surgery	−1.7	0.33	<0.01	Surgery	0.48	0.8	0.55
JCS	0.83	0.13	<0.01	JCS	1.03	0.43	0.02
Past history				Past history			
A Fib	−1.48	1.02	0.15	A Fib	1.87	0.83	0.02
CAD	0.63	0.53	0.23	CAD	−13.99	1722	0.99
Other CVD	0.17	0.42	0.69	Other CVD	−15.32	1649	0.99
PAD	−0.04	0.46	0.93	PAD	0.44	0.71	0.53
VTE	−12.04	919.33	0.99	VTE	0		
LV dysfunction	−13.02	930.55	0.99	LV dysfunction	0		
HT	−0.33	0.48	0.5	HT	14.09	1589	0.99
DM	−0.58	0.73	0.42	DM	−0.32	1.06	0.76
DLp	−0.2	0.42	0.63	DLp	−1	1.06	0.34
Genetic and others	0.58	0.78	0.46	Genetic and others	−13.84	1682	0.99
Smoking	−0.78	0.42	0.06	Smoking	−0.099	0.8	0.9
Alcohol	−0.65	0.43	0.13	Alcohol	−0.42	0.68	0.54
post-AVR	1.004	0.75	0.18	post-AVR	−13.79	1754	0.99
post-MVR	0			post-MVR	−11.24	2140	0.99
CABG	−12.14	778.45	0.99	CABG	−12.21	1995	0.99
Intervention to aortic aneurysm and/or dissection	0.019	0.63	0.98	Intervention to aortic aneurysm and/or dissection	−15.16	1769	0.99
Complication				Complication			
Major bleeding	1.62	0.39	<0.01	Major bleeding	4.17	0.81	<0.01
Infarction by aortic dissection	1.64	0.33	<0.01	Infarction by aortic dissection	2.6	0.66	<0.01
Paroxysmal A Fib	−1.34	0.53	0.01	Paroxysmal A Fib	−13.95	1622	0.99
Medication on admission				Medication on admission			
RAAS inhibitor	−0.11	0.38	0.77	RAAS inhibitor	−16.64	1879	0.99
CCB	0.52	0.36	0.14	CCB	0.14	0.71	0.85
β-blocker	−0.15	0.49	0.76	β-blocker	−0.7	1.02	0.51
diuretics	0.52	0.54	0.34	diuretics	−14.13	2151	0.99
α-blocker	1.31	0.61	0.03	α-blocker	−13.99	2021	0.99
warfarin	0.14	0.63	0.83	warfarin	−14.86	2386	0.99
DOAC	−13.12	972.53	0.99	DOAC	−13.32	2347	0.99
aspirin	0.55	0.55	0.31	aspirin	−15.46	2096	0.99
clopidogrel	−14.39	881.58	0.99	clopidogrel	−12.25	1637	0.99
cilostazol	−13.05	824.28	0.99	cilostazol	−13.7	1797	0.99
prasugrel	0			prasugrel	0		
other anti-platelet drug	1.62	1.03	0.12	other anti-platelet drug	−12.37	2454	0.99
Anti-thrombus during hospitalization				Anti-thrombus during hospitalization			
Anti-coagulant (warfarin or DOAC)	−1.64	0.6	<0.01	Anti-coagulant (warfarin or DOAC)	−15.03	2073	0.99
Anti-platelet drug (aspirin, clopidogrel, cilostazol, or prasugrel)	−2.9	1.01	<0.01	Anti-platelet drug (aspirin, clopidogrel, cilostazol, or prasugrel)	−0.31	1.08	0.78
Both anti-coagulant and anti-platelet	−15.19	859.87	0.99	Both anti-coagulant and anti-platelet	−12.29	1802	0.99
Anti-coagulant or anti-platelet	−2.17	0.53	<0.01	Anti-coagulant or anti-platelet	−0.82	1.08	0.44

SE; standard error, HR, hazard ratio, BP; blood pressure, eGFR; estimated glomerular filtration rate, JCS; Japan Coma Scale, A Fib; atrial fibrillation, CAD; coronary artery diseases, CVD; cardiovascular diseases, PAD; peripheral arterial diseases, VTE; venous thromboembolism, LV; left ventricular, HT; hypertension, DM; diabetes mellitus, DLp: dyslipidemia, AVR; aortic valve replacement, MVR; mitral valve replacement, CABG; coronary artery bypass grafting, RAAS; renin-angiotensin-aldosteron system, CCB; calcium channel blocker, DOAC; direct oral anti-coagulant. Genetic and others includes Marfan syndrome, Loeys-Dietz syndrome, and Behçet’s disease.

**Table 11:** All-cause death of anti-thrombotic agents administered during hospitalization in HOSPITALIZED patients.

Stanford type A		Administered during hospitalization		
	State on admission	no	yes	*p* value
Anti-coagulants	no	n = 307	n = 87 (a)	
	All-cause death	37 (12.1%)	3 (3.5%)	0.02
	yes	n = 8	n = 22 (b)	
	All-cause death	3 (37.5%)	0 (0.0%)	0.003
Anti-platelets	no	n = 288	n = 93 (a)	
	All-cause death	38 (13.2%)	0 (0.0%)	<0.01
	yes	n = 18	n = 25 (b)	
	All-cause death	4 (22.2%)	1 (4.0%)	0.07
Stanford type B		Administered during hospitalization		
	State on admission	no	yes	*p* value
Anti-coagulants	no	n = 206	n = 5 (a)	
	All-cause death	10 (4.9%)	0 (0.0%)	0.6
	yes	n = 4	n = 11 (b)	
	All-cause death	0 (0.0%)	0 (0.0%)	N/A
Anti-platelets	no	n = 188	n = 11 (a)	
	All-cause death	9 (4.8%)	1 (9.1%)	0.5
	yes	n = 11	n = 16 (b)	
	All-cause death	0 (0.0%)	0 (0.0%)	N/A

(a): newly administered during hospitalization, (b): continued during hospitalization, All-cause death; number (%).

## Data Availability

The data presented in this study are available on request from the corresponding author. The data are not publicly available due to privacy restrictions.

## Supplementary Materials

The following supporting information can be downloaded at: https://www.mdpi.com/article/10.3390/diagnostics12102322/s1, Supplementary Tables S1–S5: Supplementary Table S1. Cox proportional hazard model for all-cause mortality in TRANSFERRED acute aortic dissection patients (type A). Supplementary Table S2. Cox proportional hazard model for all-cause mortality in TRANSFERRED acute aortic dissection patients (type B). Supplementary Table S3. Cox proportional hazard model for all-cause mortality in HOSPITALIZED acute aortic dissection patients (type A). Supplementary Table S4. Cox proportional hazard model for all-cause mortality in HOSPITALIZED acute aortic dissection patients (type B). Supplementary Table S5. Cox proportional hazard model for all-cause mortality.
