# Supplementary material for: Use of Anti-Thrombotic Drugs and In-Hospital Mortality in Acute Aortic Dissection Patients
Source: Diagnostics (Basel). 2022 Sep 26;12(10):2322. doi: 10.3390/diagnostics12102322 (PMC9600500; doi:10.3390/diagnostics12102322)
Supplement: Supplementary file 1 [file diagnostics-12-02322-s001.zip › 20220907 Hori AAD Suppl Table S1.pdf]

Suppl Table S1. Cox proportional hazard model for all-cause mortality in TRANSFERRED acute aortic dissection patients (type A)

	Model 1			Model 2			Model 3			Model 4		
	β	SE	p-value	β	SE	p-value	β	SE	p-value	β	SE	p-value
Age	0.01	0.01	0.23	-	-	-	-	-	-	-	-	-
Sex	-0.02	0.23	0.94	-	-	-	-	-	-	-	-	-
Systolic BP	-0.02	<0.01	**<0.01**	-0.02	0.004	**<0.01**	-	-	-	-	-	-
Diastolic BP	-0.01	0.008	0.19	-0.01	0.008	0.17	0.004	0.01	0.76	0.01	0.01	0.51
Heart rate	0.011	0.006	0.05	0.01	0.006	0.07	-0.003	0.007	0.68	-0.004	0.007	0.57
eGFR	-0.028	0.006	**<0.01**	-0.03	0.006	**<0.01**	-0.027	0.007	**<0.01**	-	-	-
Surgery	-2.39	0.25	**<0.01**	-2.38	0.25	**<0.01**	-	-	-	-	-	-
JCS	0.86	0.1	**<0.01**	0.85	0.1	**<0.01**	0.54	0.14	**<0.01**	0.4	0.17	**0.01**
Past history												
A Fib	-1.05	0.72	0.15	-1.21	0.72	0.1	-0.8	0.74	0.28	-	-	-
CAD	0.76	0.4	0.06	0.7	0.4	0.09	0.7	0.49	0.15	-	-	-
Other CVD	0.005	0.36	0.99	-0.08	0.37	0.83	-0.08	0.4	0.84	-	-	-
PAD	0.16	0.35	0.64	0.05	0.36	0.89	0.08	0.41	0.85	0.07	0.43	0.87
VTE	-12.01	744.56	0.99	-12.06	752.05	0.99	-11.5	771.4	0.98	-12.3	1284	0.99
LV dysfunction	-13.03	789.73	0.99	-12.99	788.9	0.99	-12.4	1015	0.99	-	-	-
HT	-0.8	0.31	**0.01**	-0.88	0.32	**<0.01**	-0.26	0.39	0.5	-0.55	0.44	0.21
DM	-0.55	0.59	0.35	-0.58	0.59	0.33	-	-	-	-	-	-
DLp	-0.23	0.33	0.49	-0.2	0.34	0.56	-	-	-	-	-	-
Genetic and others	0.57	0.62	0.35	0.71	0.63	0.26	0.53	0.87	0.55	1.35	0.87	0.12
Smoking	-0.64	0.31	**0.04**	-0.86	0.36	**0.02**	-	-	-	-	-	-
Alcohol	-0.68	0.34	**0.04**	-0.8	0.38	**0.03**	-0.67	0.4	0.1	-0.56	0.46	0.22
post-AVR	1.02	0.59	0.09	1.01	0.61	0.1	1.3	0.63	**0.04**	1.69	0.76	**0.03**
post-MVR	0	.	.	0	.	.	0	.	.	0	.	.
CABG	-12.01	627.8	0.98	-12.24	633.99	0.98	-11.5	822.9	0.98	-13	1317	0.99
Intervention to aortic aneurysm and/or dissection	0.27	0.47	0.57	0.18	0.49	0.72	0.73	0.51	0.15	0.61	0.53	0.25
Complication												
Major bleeding	1.52	0.32	**<0.01**	1.48	0.32	**<0.01**	1.86	0.37	**<0.01**	2.3	0.43	**<0.01**
Infarction by aortic dissection	1.54	0.27	**<0.01**	1.52	0.27	**<0.01**	1.47	0.32	**<0.01**	1.2	0.36	**<0.01**
Paroxymal A Fib	-1.66	0.52	**<0.01**	-1.75	0.52	**<0.01**	-1.28	0.53	**0.02**	-1.6	0.62	**0.01**
Medication on admission												
RAAS inhibitor	-0.06	0.31	0.85	-0.14	0.31	0.66	0.09	0.34	0.79	-0.12	0.37	0.75
CCB	0.36	0.29	0.22	0.29	0.29	0.33	0.85	0.34	**0.01**	0.88	0.39	**0.02**
β-blocker	0.04	0.39	0.93	-0.05	0.39	0.89	0.34	0.44	0.44	0.03	0.55	0.96
diuretics	0.6	0.44	0.17	0.48	0.44	0.27	1.17	0.46	**0.01**	1.19	0.52	**0.02**
α-blocker	1.2	0.52	**0.02**	1.16	0.52	0.03	1.23	0.54	**0.02**	0.98	0.59	0.09
warfarin	0.16	0.53	0.77	-0.002	0.54	0.99	0.1	0.56	0.85	0.34	0.78	0.66
DOAC	-13.03	787.37	0.99	-13.24	796.32	0.99	-13.1	1146	0.99	-12.8	2322	0.99
aspirin	0.8	0.41	0.05	0.75	0.42	0.07	0.85	0.5	0.09	0.47	0.68	0.49
clopidogrel	-14.17	771.42	0.99	-14.35	776.75	0.99	-12.8	797.1	0.98	-13.4	882.2	0.99
cilostazol	-13.03	673.45	0.98	-13.12	675.96	0.98	-12.6	796.4	0.99	-13.7	1306	0.99
prasugrel	0	.	.	0	.	.	0	.	.	0	.	.
other anti-platelet drug	1.18	1.01	0.24	1.14	1.02	0.27	1.36	1.12	0.23	1.38	1.16	0.23
Anti-coagulant (warfarin or DOAC)	-0.33	0.52	0.52	-0.48	0.53	0.36	-0.07	0.54	0.9	0.25	0.73	0.73
Anti-platelet drug (aspirin, clopidogrel, cilostazol, or prasugrel)	-0.02	0.38	0.96	-0.11	0.38	0.78	0.46	0.46	0.32	0.14	0.61	0.82
Both anti-coagulant and anti-platelet	0.55	0.72	0.44	0.49	0.72	0.5	0.62	0.75	0.4	0.11	0.92	0.91
Anti-coagulant or anti-platelet	-0.26	0.34	0.45	-0.41	0.36	0.25	0.14	0.41	0.74	0.24	0.58	0.68

Model 1 : Unadjusted

Model 2 : Adjusted for age and sex

Model 3 : Adjusted for age, sex, surgery, systolic BP, DLp, DM and smoking

Model 4 : Model 3 + history of A fib, CAD, other CVD and LV dysfunction

SE; standard error, HR, hazard ratio, BP; blood pressure, eGFR; estimated glomerular filtration rate, A Fib; atrial fibrillation, CAD; coronary artery diseases, CVD; cardiovascular diseases, PAD; peripheral arterial diseases, VTE; venous thromboembolism, LV; left ventricular, HT; hypertension, DM; diabetes mellitus, DLp: dyslipidemia, AVR; aortic valve replacement, MVR; mitral valve replacement, CABG; coronary artery bypass grafting, RAAS; renin-angiotensin-aldosteron system, CCB; calcium channel blocker, DOAC; direct oral anti-coagulant.

Genetic and others includes Marfan syndrome, Loeys-Dietz syndrome, and Behçet's disease.
