# Supplementary material for: Use of Anti-Thrombotic Drugs and In-Hospital Mortality in Acute Aortic Dissection Patients
Source: Diagnostics (Basel). 2022 Sep 26;12(10):2322. doi: 10.3390/diagnostics12102322 (PMC9600500; doi:10.3390/diagnostics12102322)
Supplement: Supplementary file 1 [file diagnostics-12-02322-s001.zip › 20220907 Hori AAD Suppl Table S2.pdf]

**Suppl Table S2. Cox proportional hazard model for all-cause mortality in TRANSFERRED acute aortic dissection patients (type B)**

| | Model 1 | | | Model 2 | | | Model 3 | | | Model 4 | | |
|---|---|---|---|---|---|---|---|---|---|---|---|---|
| | β | SE | p-value | β | SE | p-value | β | SE | p-value | β | SE | p-value |
| Age | 0.0005 | 0.02 | 0.98 | - | - | - | - | - | - | - | - | - |
| Sex | -0.64 | 0.65 | 0.32 | - | - | - | - | - | - | - | - | - |
| Systolic BP | -0.03 | 0.01 | **0.02** | -0.03 | 0.01 | **0.01** | - | - | - | - | - | - |
| Diastolic BP | -0.02 | 0.02 | 0.24 | -0.02 | 0.02 | 0.18 | 0.006 | 0.024 | 0.8 | 0.01 | 0.03 | 0.63 |
| Heart rate | 0.03 | 0.01 | **0.04** | 0.03 | 0.02 | 0.05 | 0.04 | 0.02 | **0.02** | 0.02 | 0.02 | 0.25 |
| eGFR | -0.02 | 0.01 | **0.04** | -0.02 | 0.01 | **0.02** | -0.03 | 0.01 | **0.04** | | | |
| Surgery | 0.77 | 0.59 | 0.19 | 0.85 | 0.59 | 0.15 | | | | | | |
| JCS | 1.23 | 0.25 | **<0.01** | 1.23 | 0.26 | **<0.01** | 0.86 | 0.42 | **0.04** | 0.55 | 0.58 | 0.35 |
| Past history | | | | | | | | | | | | |
| A Fib | 1.93 | 0.67 | **<0.01** | 2.05 | 0.69 | **<0.01** | 2.1 | 0.72 | **0.004** | - | - | - |
| CAD | -14.08 | 1484 | 0.99 | -14 | 1480 | 0.99 | -13.9 | 1557 | 0.99 | - | - | - |
| Other CVD | 0.18 | 0.77 | 0.81 | 0.16 | 0.78 | 0.83 | 0.03 | 0.8 | 0.97 | - | - | - |
| PAD | 0.47 | 0.6 | 0.43 | 0.5 | 0.61 | 0.41 | 0.42 | 0.63 | 0.51 | 0.77 | 0.75 | 0.31 |
| VTE | 0 | . | . | 0 | . | . | 0 | . | . | 0 | . | . |
| LV dysfunction | 0 | . | . | 0 | . | . | 0 | . | . | - | - | - |
| HT | 14.09 | 1400 | 0.99 | 14.07 | 1400 | 0.99 | 14.4 | 1435 | 0.99 | 14.5 | 2278 | 0.99 |
| DM | 0.12 | 0.77 | 0.87 | 0.17 | 0.77 | 0.82 | - | - | - | - | - | - |
| DLp | -0.04 | 0.66 | 0.95 | -0.02 | 0.66 | 0.98 | - | - | - | - | - | - |
| Genetic and others | -14.07 | 1509 | 0.99 | -14.11 | 1513 | 0.99 | -14 | 1560 | 0.99 | -14.6 | 2338 | 0.99 |
| Smoking | 0.21 | 0.6 | 0.72 | -0.01 | 0.7 | 0.99 | - | - | - | - | - | - |
| Alcohol | -0.2 | 0.57 | 0.73 | -0.39 | 0.6 | 0.52 | -0.25 | 0.65 | 0.71 | -0.35 | 0.76 | 0.65 |
| post-AVR | -14.09 | 1577 | 0.99 | -14.02 | 1587 | 0.99 | -14.2 | 1695 | 0.99 | -14.5 | 1898 | 0.99 |
| post-MVR | -11.01 | 1501 | 0.99 | -11.13 | 1515 | 0.99 | -11.4 | 1646 | 0.99 | -1.43 | 10117 | 0.99 |
| CABG | -12.01 | 1543 | 0.99 | -12.16 | 1558 | 0.99 | -11.8 | 1685 | 0.99 | -1.02 | 6215 | 0.99 |
| Intervention to aortic aneurysm and/or dissection | -0.45 | 1.04 | 0.67 | -0.41 | 1.05 | 0.7 | -0.5 | 1.1 | 0.64 | -15.6 | 2271 | 0.99 |
| Complication | | | | | | | | | | | | |
| Major bleeding | 3.87 | 0.59 | **<0.01** | 4.24 | 0.71 | **<0.01** | 4.77 | 0.94 | **<0.01** | 4.42 | 1.11 | **<0.01** |
| Infarction by aortic dissection | 2.04 | 0.57 | **<0.01** | 2.04 | 0.57 | **<0.01** | 2.12 | 0.61 | **<0.01** | 2.16 | 0.72 | **<0.01** |
| Paroxymal A Fib | -14.09 | 1435 | 0.99 | -13.95 | 1435 | 0.99 | -14 | 1492 | 0.99 | -14.1 | 2302 | 0.99 |
| Medication on admission | | | | | | | | | | | | |
| RAAS inhibitor | -16.67 | 1711 | 0.99 | -16.6 | 1710 | 0.99 | -16.7 | 1742 | 0.99 | -17.6 | 1910 | 0.99 |
| CCB | 0.14 | 0.61 | 0.82 | 0.33 | 0.63 | 0.6 | 0.19 | 0.69 | 0.78 | -0.17 | 0.84 | 0.84 |
| β-blocker | -0.2 | 0.78 | 0.8 | -0.05 | 0.78 | 0.94 | -0.33 | 0.82 | 0.69 | -1.39 | 1.17 | 0.24 |
| diuretics | 1.9 | 0.79 | **0.02** | 1.83 | 0.79 | **0.02** | 1.88 | 0.87 | **0.03** | 0.91 | 1.14 | 0.43 |
| α-blocker | -14.05 | 1838 | 0.99 | -13.97 | 1835 | 0.99 | -14.1 | 1840 | 0.99 | -15 | 3093 | 0.99 |
| warfarin | 0.4 | 1.06 | 0.71 | 0.7 | 1.07 | 0.51 | 0.64 | 1.09 | 0.56 | -1.05 | 1.59 | 0.51 |
| DOAC | -13.02 | 2047 | 0.99 | -13.25 | 2020 | 0.99 | -13.2 | 2205 | 0.99 | -15.5 | 6501 | 0.99 |
| aspirin | -15.16 | 1903 | 0.99 | -15.33 | 1875 | 0.99 | -15.5 | 1957 | 0.99 | -16.7 | 2867 | 0.99 |
| clopidogrel | -13.04 | 2043 | 0.99 | -13.26 | 2052 | 0.99 | -13.2 | 1963 | 0.99 | -13.8 | 6529 | 0.99 |
| cilostazol | -13.05 | 1571 | 0.99 | -13.68 | 1616 | 0.99 | -14.7 | 2098 | 0.99 | 0 | . | . |
| prasugrel | 0 | . | . | 0 | . | . | 0 | . | . | 0 | . | . |
| other anti-platelet drug | -12.01 | 2086 | 0.99 | -12.41 | 2152 | 0.99 | -11.4 | 1486 | 0.99 | -15.3 | 8158 | 0.99 |
| Anti-coagulant (warfarin or DOAC) | -0.12 | 1.04 | 0.91 | 0.05 | 1.05 | 0.96 | 0.24 | 1.08 | 0.83 | -0.86 | 1.45 | 0.55 |
| Anti-platelet drug (aspirin, clopidogrel, cilostazol, or prasugrel) | -15.19 | 1457 | 0.99 | -15.19 | 1449 | 0.99 | -15.1 | 1556 | 0.99 | -16.8 | 2309 | 0.99 |
| Both anti-coagulant and anti-platelet | -13.03 | 1218 | 0.99 | -13.01 | 1217 | 0.99 | -13.1 | 1428 | 0.99 | -14.9 | 2180 | 0.99 |
| Anti-coagulant or anti-platelet | -1.02 | 1.04 | 0.33 | -0.94 | 1.05 | 0.37 | -0.68 | 1.1 | 0.54 | -2.6 | 1.8 | 0.15 |

Model 1 : Unadjusted

Model 2 : Adjusted for age and sex

Model 3 : Adjusted for age, sex, surgery, systolic BP, DLp, DM and smoking

Model 4 : Model 3 + history of A fib, CAD, other CVD and LV dysfunction

SE; standard error, HR, hazard ratio, BP; blood pressure, eGFR; estimated glomerular filtration rate, A Fib; atrial fibrillation, CAD; coronary artery diseases, CVD; cardiovascular diseases, PAD; peripheral arterial diseases, VTE; venous thromboembolism, LV; left ventricular, HT; hypertension, DM; diabetes mellitus, DLp: dyslipidemia, AVR; aortic valve replacement, MVR; mitral valve replacement, CABG; coronary artery bypass grafting, RAAS; renin-angiotensin-aldosteron system, CCB; calcium channel blocker, DOAC; direct oral anti-coagulant.

Genetic and others includes Marfan syndrome, Loeys-Dietz syndrome, and Behçet's disease.
