# Supplementary material for: Use of Anti-Thrombotic Drugs and In-Hospital Mortality in Acute Aortic Dissection Patients
Source: Diagnostics (Basel). 2022 Sep 26;12(10):2322. doi: 10.3390/diagnostics12102322 (PMC9600500; doi:10.3390/diagnostics12102322)
Supplement: Supplementary file 1 [file diagnostics-12-02322-s001.zip › 20220907 Hori AAD Suppl Table S3.pdf]

Suppl Table S3. Cox proportional hazard model for all-cause mortality in HOSPITALIZED acute aortic dissection patients (type A)

	Model 1			Model 2			Model 3			Model 4		
	β	SE	p-value	β	SE	p-value	β	SE	p-value	β	SE	p-value
Age	0.006	0.01	0.65	-	-	-	-	-	-	-	-	-
Sex	-0.11	0.31	0.72	-	-	-	-	-	-	-	-	-
Systolic BP	-0.02	0.006	**<0.01**	-0.02	0.006	**<0.01**	-	-	-	-	-	-
Diastolic BP	-0.005	0.01	0.63	-0.005	0.01	0.61	0.021	0.015	0.16	0.02	0.016	0.23
Heart rate	0.01	0.008	0.11	0.012	0.008	0.12	-0.0014	0.0081	0.87	-0.004	0.0088	0.65
eGFR	-0.028	0.007	**<0.01**	-0.028	0.007	**<0.01**	-0.027	0.0081	**<0.01**			
Surgery	-1.7	0.33	**<0.01**	-1.7	0.33	**<0.01**	-	-	-	-	-	-
JCS	0.82	0.13	**<0.01**	0.83	0.13	**<0.01**	0.73	0.16	**<0.01**	0.61	0.18	**<0.01**
Past history												
A Fib	-1.34	1.01	0.19	-1.48	1.02	0.15	-1.29	1.03	0.21	-	-	-
CAD	0.67	0.53	0.21	0.63	0.53	0.23	0.77	0.55	0.16	-	-	-
Other CVD	0.22	0.42	0.6	0.17	0.42	0.69	0.15	0.43	0.73	-	-	-
PAD	0.04	0.45	0.93	-0.04	0.46	0.93	0.072	0.47	0.88	-0.012	0.49	0.98
VTE	-12.01	915.23	0.99	-12.04	919.33	0.99	-12.3	1167	0.99	-13.3	2050	0.99
LV dysfunction	-13.04	930.19	0.99	-13.02	930.55	0.99	-12.1	0.5	0.73	-	-	-
HT	-0.28	0.48	0.56	-0.33	0.48	0.5	-0.18	0.5	0.73	-0.46	0.53	0.39
DM	-0.56	0.73	0.44	-0.58	0.73	0.42	-	-	-	-	-	-
DLp	-0.23	0.42	0.58	-0.2	0.42	0.63	-	-	-	-	-	-
Genetic and others	0.47	0.77	0.54	0.58	0.78	0.46	0.53	0.87	0.55	1.37	0.88	0.12
Smoking	-0.52	0.36	0.15	-0.78	0.42	0.06	-	-	-	-	-	-
Alcohol	-0.52	0.38	0.18	-0.65	0.43	0.13	-0.58	0.45	0.2	-0.26	0.49	0.59
post-AVR	1.03	0.73	0.16	1.004	0.75	0.18	1.64	0.78	**0.03**	1.7	0.84	**0.04**
post-MVR	0 .	.		0 .	.		0 .	.		0 .	.	
CABG	-12.01	776.4	0.99	-12.14	778.45	0.99	-12.1	1153	0.99	-13.9	2048	0.99
Intervention to aortic aneurysm and/or dissection	0.1	0.61	0.87	0.019	0.63	0.98	0.44	0.64	0.5	0.32	0.67	0.63
Complication												
Major bleeding	1.66	0.38	**<0.01**	1.62	0.39	**<0.01**	1.99	0.44	**<0.01**	2.24	0.48	**<0.01**
Infarction by aortic dissection	1.63	0.33	**<0.01**	1.64	0.33	**<0.01**	1.68	0.36	**<0.01**	1.45	0.39	**<0.01**
Paroxymal A Fib	-1.27	0.53	**0.02**	-1.34	0.53	**0.01**	-1.16	0.54	**0.03**	-1.49	0.62	**0.02**
Medication on admission												
RAAS inhibitor	-0.073	0.38	0.85	-0.11	0.38	0.77	-0.077	0.4	0.85	-0.16	0.42	0.71
CCB	0.54	0.35	0.12	0.52	0.36	0.14	0.85	0.38	**0.03**	0.81	0.42	0.06
β-blocker	-0.1	0.49	0.84	-0.15	0.49	0.76	0.15	0.51	0.77	-0.016	0.59	0.98
diuretics	0.58	0.54	0.28	0.52	0.54	0.34	1.26	0.57	**0.03**	0.97	0.63	0.12
α-blocker	1.32	0.61	**0.03**	1.31	0.61	**0.03**	1.66	0.65	**0.01**	1.7	0.67	**0.01**
warfarin	0.23	0.61	0.71	0.14	0.63	0.83	0.13	0.65	0.85	0.38	0.84	0.65
DOAC	-13.03	969.9	0.99	-13.12	972.53	0.99	-12.8	1184	0.99	-12.3	2183	0.99
aspirin	0.61	0.54	0.26	0.55	0.55	0.31	0.82	0.56	0.15	0.68	0.69	0.32
clopidogrel	-14.21	884.34	0.99	-14.39	881.58	0.99	-13.5	1183	0.99	-14.5	1343	0.99
cilostazol	-13.04	823.72	0.99	-13.05	824.28	0.99	-13.2	1208	0.99	-13.4	1289	0.99
prasugrel	0 .	.		0 .	.		0 .	.		0 .	.	
other anti-platelet drug	1.69	1.02	0.1	1.62	1.03	0.12	2.24	1.15	0.05	2.38	1.23	0.05
Anti-thrombus during hospitalization												
Anti-coagulant (warfarin or DOAC)	-1.62	0.6	**<0.01**	-1.64	0.6	**<0.01**	-1.58	0.62	**0.01**	-1.47	0.63	**0.02**
Anti-platelet drug (aspirin, clopidogrel, cilostazol, or prasugrel)	-2.88	1.01	**<0.01**	-2.9	1.01	**<0.01**	-2.58	1.02	**0.01**	-2.74	1.03	**0.008**
Both anti-coagulant and anti-platelet	-15.2	860.56	0.99	-15.19	859.87	0.99	-15.4	1040	0.99	-15.8	1145	0.99
Anti-coagulant or anti-platelet	-2.12	0.53	**<0.01**	-2.17	0.53	**<0.01**	-2	0.54	**<0.01**	-1.95	0.56	**<0.01**

Model 1 : Unadjusted

Model 2 : Adjusted for age and sex

Model 3 : Adjusted for age, sex, surgery, systolic BP, DLp, DM and smoking

Model 4 : Model 3 + history of A fib, CAD, other CVD and LV dysfunction

SE; standard error, HR, hazard ratio, BP; blood pressure, eGFR; estimated glomerular filtration rate, A Fib; atrial fibrillation, CAD; coronary artery diseases, CVD; cardiovascular diseases, PAD; peripheral arterial diseases, VTE; venous thromboembolism, LV; left ventricular, HT; hypertension, DM; diabetes mellitus, DLp: dyslipidemia, AVR; aortic valve replacement, MVR; mitral valve replacement, CABG; coronary artery bypass grafting, RAAS; renin-angiotensin-aldosteron system, CCB; calcium channel blocker, DOAC; direct oral anti-coagulant.

Genetic and others includes Marfan syndrome, Loeys-Dietz syndrome, and Behçet's disease.
