# Supplementary material for: Use of Anti-Thrombotic Drugs and In-Hospital Mortality in Acute Aortic Dissection Patients
Source: Diagnostics (Basel). 2022 Sep 26;12(10):2322. doi: 10.3390/diagnostics12102322 (PMC9600500; doi:10.3390/diagnostics12102322)
Supplement: Supplementary file 1 [file diagnostics-12-02322-s001.zip › 20220907 Hori AAD Suppl Table S4.pdf]

Suppl Table S4. Cox proportional hazard model for all-cause mortality in HOSPITALIZED acute aortic dissection patients (type B)

	Model 1			Model 2			Model 3			Model 4		
	**β**	**SE**	**p-value**	**β**	**SE**	**p-value**	**β**	**SE**	**p-value**	**β**	**SE**	**p-value**
Age	0.004	0.025	0.88	-	-	-	-	-	-	-	-	-
Sex	-0.65	0.79	0.41	-	-	-	-	-	-	-	-	-
Systolic BP	-0.011	0.014	0.4	-0.012	0.014	0.37	-	-	-	-	-	-
Diastolic BP	-0.002	0.018	0.9	-0.005	0.019	0.79	0.013	0.027	0.63	0.026	0.03	0.39
Heart rate	0.033	0.016	**0.04**	0.035	0.018	0.05	0.038	0.019	**0.04**	0.029	0.022	0.19
eGFR	-0.022	0.012	0.08	-0.025	0.013	0.05	-0.025	0.014	0.08	-	-	-
Surgery	0.38	0.8	0.63	0.48	0.8	0.55	-	-	-	-	-	-
JCS	1.04	0.42	**0.01**	1.03	0.43	**0.02**	0.9	0.52	0.09	0.86	0.71	0.23
Past history												
A Fib	1.79	0.8	**0.03**	1.87	0.83	**0.02**	1.89	0.88	**0.03**	-	-	-
CAD	-14.08	1721	0.99	-13.99	1722	0.99	-13.5	1780	0.99	-	-	-
Other CVD	-15.23	1650	0.99	-15.32	1649	0.99	-15.4	1609	0.99	-	-	-
PAD	0.44	0.69	0.53	0.44	0.71	0.53	0.56	0.72	0.44	1.39	0.84	0.09
VTE	0 .	.		0 .	.		0 .	.		0 .	.	
LV dysfunction	0 .	.		0 .	.		0 .	.		-	-	-
HT	14.1	1594	0.99	14.09	1589	0.99	14.59	1621	0.99	16.68	4979	0.99
DM	-0.38	1.06	0.72	-0.32	1.06	0.76	-	-	-	-	-	-
DLp	-1.02	1.05	0.33	-1	1.06	0.34	-	-	-	-	-	-
Genetic and others	-14.07	1681	0.99	-13.84	1682	0.99	-13.99	1749	0.99	-16	5052	0.99
Smoking	0.26	0.69	0.71	-0.099	0.8	0.9	-	-	-	-	-	-
Alcohol	-0.15	0.65	0.82	-0.42	0.68	0.54	-0.29	0.73	0.69	-0.64	0.86	0.46
post-AVR	-14.1	1749	0.99	-13.79	1754	0.99	-13.8	1788	0.99	-16.1	4672	0.99
post-MVR	-11.01	2104	0.99	-11.24	2140	0.99	-11.7	2274	0.99	-2.72	36423	0.99
CABG	-12.01	1962	0.99	-12.21	1995	0.99	-11.1	2106	0.99	-0.2	20754	1
Intervention to aortic aneurysm and/or dissection	-15.21	1776	0.99	-15.16	1769	0.99	-15	1771	0.99	-15.5	3049	0.99
Complication												
Major bleeding	3.82	0.68	**<0.01**	4.17	0.81	**<0.01**	4.96	1.12	**<0.01**	4.87	1.3	**<0.01**
Infarction by aortic dissection	2.58	0.66	**<0.01**	2.6	0.66	**<0.01**	2.93	0.74	**<0.01**	2.55	0.82	**<0.01**
Paroxymal A Fib	-14.1	1623	0.99	-13.95	1622	0.99	-13.7	1586	0.99	-16.1	5844	0.99
Medication on admission												
RAAS inhibitor	-16.67	1876	0.99	-16.64	1879	0.99	-16.8	1837	0.99	-17.88	2867	0.99
CCB	-0.014	0.69	0.98	0.14	0.71	0.85	0.079	0.76	0.92	0.05	0.85	0.95
β-blocker	-0.81	1.06	0.44	-0.7	1.02	0.51	-0.98	1.09	0.37	-18.1	3078	0.99
diuretics	-14.05	2135	0.99	-14.13	2151	0.99	-14.4	2232	0.99	-17.7	6448	0.99
α-blocker	-14.05	2021	0.99	-13.99	2021	0.99	-13.8	2025	0.99	-16.2	7084	0.99
warfarin	-15.13	2435	0.99	-14.86	2386	0.99	-14.8	2284	0.99	-15.9	2732	0.99
DOAC	-13.02	2372	0.99	-13.32	2347	0.99	-12.7	2425	0.99	-17.7	46153	0.99
aspirin	-15.17	2114	0.99	-15.46	2096	0.99	-15.2	2221	0.99	-17.9	4184	0.99
clopidogrel	-12.03	1628	0.99	-12.25	1637	0.99	-13.1	2527	0.99	-15.7	18002	0.99
cilostazol	-13.06	1757	0.99	-13.7	1797	0.99	-13.3	2184	0.99	0 .	.	
prasugrel	0 .	.		0 .	.		0 .	.		0 .	.	
other anti-platelet drug	-12.01	2395	0.99	-12.37	2454	0.99	-11.6	2603	0.99	-18.2	35679	0.99
Anti-thrombus during hospitalization												
Anti-coagulant (warfarin or DOAC)	-15.13	2087	0.99	-15.03	2073	0.99	-14.9	2056	0.99	-16.2	3448	0.99
Anti-platelet drug (aspirin, clopidogrel, cilostazol, or prasugrel)	-0.38	1.06	0.72	-0.31	1.08	0.78	-0.04	1.11	0.97	0.34	1.17	0.77
Both anti-coagulant and anti-platelet	-12.01	1770	0.99	-12.29	1802	0.99	-11.23	1907	0.99	-15.3	19601	0.99
Anti-coagulant or anti-platelet	-0.91	1.06	0.39	-0.82	1.08	0.44	-0.62	1.09	0.57	-0.46	1.13	0.68

Model 1 : Unadjusted

Model 2 : Adjusted for age and sex

Model 3 : Adjusted for age, sex, surgery, systolic BP, DLp, DM and smoking

Model 4 : Model 3 + history of A fib, CAD, other CVD and LV dysfunction

SE; standard error, HR, hazard ratio, BP; blood pressure, eGFR; estimated glomerular filtration rate, A Fib; atrial fibrillation, CAD; coronary artery diseases, CVD; cardiovascular diseases, PAD; peripheral arterial diseases, VTE; venous thromboembolism, LV; left ventricular, HT; hypertension, DM; diabetes mellitus, DLp: dyslipidemia, AVR; aortic valve replacement, MVR; mitral valve replacement, CABG; coronary artery bypass grafting, RAAS; renin-angiotensin-aldosteron system, CCB; calcium channel blocker, DOAC; direct oral anti-coagulant.

Genetic and others includes Marfan syndrome, Loeys-Dietz syndrome, and Behçet's disease.
